# Design and synthesis of highly active MoVTeNb-oxides for ethane oxidative dehydrogenation

**DOI:** 10.1038/s41467-019-11940-0

**Published:** 2019-09-05

**Authors:** Daniel Melzer, Gerhard Mestl, Klaus Wanninger, Yuanyuan Zhu, Nigel D. Browning, Maricruz Sanchez-Sanchez, Johannes A. Lercher

**Affiliations:** 1 Department Chemie & Catalysis Research Center, TU München, Lichtenbergstr. 4, D-85747 Garching, Germany; 2 Clariant Produkte (Deutschland) GmbH, Waldheimer Str. 13, D-83502 Bruckmühl, Germany; 3 Department of Materials Science and Engineering, Institute of Materials Science, University of Connecticut, Storrs, CT 06269 USA; 4 Institute for Integrated Catalysis, Pacific Northwest National Laboratory, Richland, WA 99352 USA; 5 Imaging Center at Liverpool (ICaL), School of Engineering & School of Physical Sciences, University of Liverpool, 506 Brodie Tower, Liverpool, L69 3GQ UK

**Keywords:** Catalyst synthesis, Heterogeneous catalysis, Chemical engineering

## Abstract

Ethane oxidative dehydrogenation (ODH) is an alternative route for ethene production. Crystalline M1 phase of Mo-V mixed metal oxide is an excellent catalyst for this reaction. Here we show a hydrothermal synthesis method that generates M1 phases with high surface areas starting from poorly soluble metal oxides. Use of organic additives allows control of the concentration of metals in aqueous suspension. Reactions leading to crystalline M1 take place at 190 °C, i.e., approximately 400 °C lower than under current synthesis conditions. The evolution of solvated polyoxometalate ions and crystalline phases in the solid is monitored by spectroscopies. Catalysts prepared by this route show higher ODH activity compared to conventionally prepared catalysts. The higher activity is due not only to the high specific surface area but also to the corrugated lateral termination of the M1 crystals, as seen by atomic resolution electron microscopy, exposing a high concentration of catalytically active sites.

## Abstract (web summary)

Crystalline M1 phase of Mo-V-Te-Nb mixed oxide is an excellent catalyst for ethane oxidative dehydrogenation to ethene. Here, the authors show a method that synthesizes highly active materials by generating M1 crystals with corrugated terminations, thus exposing a large concentration of active sites.

## Introduction

Oxidative dehydrogenation of ethane (ODH-E) over M1-type MoV(Te,Nb)-mixed metal oxides has emerged as one of the most promising routes to on-purpose conversion of widely available, but locally dispersed ethane from shale gas resources^1,2^. Such conversion processes must combine single pass ethene yields exceeding 60% with minimal selectivities to carbon oxides^3^. Mo-V-mixed oxide catalysts approach these criteria when crystallized in pure M1 phase with formulations that include Te and Nb^4^. However, potential industrial realization still requires higher ethane conversion rates and selectivities than those achieved to date^3^. The weight normalized conversion rates are currently limited by the presence of catalytically inactive crystalline and amorphous phases and by the relatively low specific surface area of the active M1 phase (5–15 m^2^ g^−1^).

**Fig. 1:**
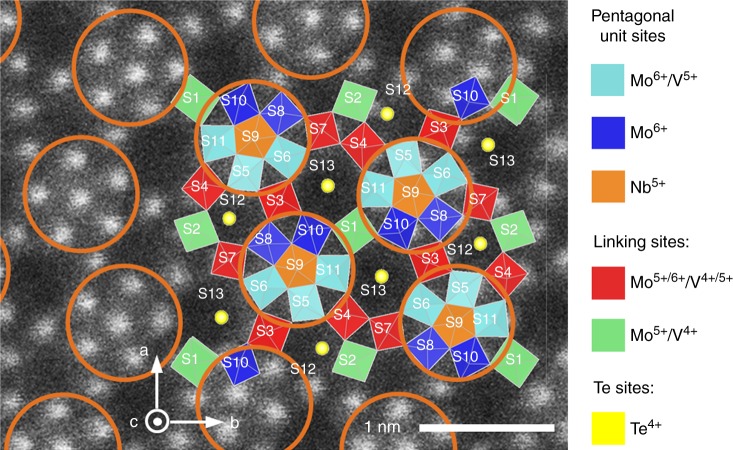
Schematic view of M1 crystal in the [001] direction. Overlay of 2 × 2 unit cells representation with a high-angle annular dark-field scanning transmission electron microscopy (HAADF-STEM) image of M1 basal plane. Different colors of crystallographic sites indicate occupancy by different metals and oxidation states. To guide the eye, pentagonal Nb-centered M_6_O_21_ building blocks have been marked with orange circles

The activity of M1 phase has been originally attributed to sites that are only exposed in the minority {001} plane of the catalyst particles^5–8^. The surface of the crystalline {001} plane (Fig. 1) consists of pentagonal Nb-centered pillars surrounded by edge and corner-sharing MoO_6_ and VO_6_ octahedra. These pentagonal units are connected by further metal-oxygen octahedra forming hexagonal and heptagonal channels, which are partially occupied by Te. The catalytic site able to abstract the first ethane hydrogen is a V^5+^ = O group in an ensemble formed by S2, S4, and S7 metal positions of the M1 structure (Fig. 1)^9–12^. Lately, hydrogen abstraction activity has also been speculated to benefit from confinement of hydrocarbons in the heptagonal channels of M1-type MoVTeNbO_x_^13^ and MoVO_x_^14,15^. We have shown unequivocally, however, that catalytic sites can also be present at the side walls of the catalyst crystallites, if suitably terminated^16^. The concentration of such sites is limited by conventional synthesis strategies of M1-type oxides in which hydrothermal synthesis, evaporation or spray drying leads to X-ray amorphous precursors that are crystallized by a thermal post-treatment between 550 and 650 °C^17–23^.

Strategies to increase the activity aim to increase the fraction of terminating surfaces that contain high concentrations of active sites. In the case of M1 phase, an obvious strategy would be to limit growth of the crystals along the *c*-axis to increase the relative contribution of {001} planes. However, bronze-type materials like M1 phase show a strong preference to grow along the *c*-axis and, thus, inhibiting crystal growth in the [001] direction would likely hamper the overall crystallization. On the other hand, growth with open lateral terminations as those shown by rod-like particles leads conceptually to a significant enhancement of catalytic activity, without negatively affecting selectivity^16^.

We report here a different strategy for the synthesis of M1 phase with crystallites having a highly active surface termination of the side walls of the rod-like structures. This is achieved by enabling the direct formation of quaternary (Mo-V-Te-Nb) M1 phase without a high-temperature crystallization step. We achieve this by using additives to control the formation of polyoxometalate clusters such as {Mo_72_V_30_} in solution, which have been shown to be intermediates in the formation of the M1-phase of MoV mixed metal oxides^24,25^. In contrast to the strategies using hydrothermal syntheses routes with well-soluble metal complexes and salts, we minimize the concentration of the oxide-forming constituents in solution. This allows on one hand a more directed selective synthesis, and on the other hand it enables growth of highly active surfaces in the a–b direction. This is achieved by generating an environment in which high activity coefficients of the metal-hydroxo precursors are induced by additives.

## Results

### Towards a tailored synthesis of highly active MoVTeNb oxides

The central hypothesis for direct synthesis of MoVTeNb oxide M1 phase was to generate reactive precursors at rates that are equal to the rate of (hydrothermal) crystallization. To achieve a low rate of production of precursors we use poorly soluble metal oxide precursors, i.e., MoO_3_, V_2_O_5_, TeO_2_ and Nb_2_O_5_. Their rate of dissolution was adjusted by addition of citric acid, monoethylene glycol and oxalic acid as complexing agents. The presence of these additives is required for observable rates of crystallization (Supplementary Fig. 1).

Varying the nominal relative concentrations of the metal cations, i.e., Mo:V:Te:Nb = 1:0.22–0.30:0–0.18:0–0.18, led to widely varying materials. Let us first look at a series of samples with constant Te and Nb content (Te/Mo = Nb/Mo = 0.18) and different V/Mo ratios. The starting nominal composition was chosen to allow a direct comparison with the M1 syntheses in ref. ^26^. For the range of V/Mo ratios explored, only short-range ordering of the dried mixed metal oxides was observed. The X-ray diffraction (XRD) results (see Supplementary Fig. 2 for details) show broad reflections characteristic of nanocrystals, frequently reported together with unordered domains in M1 crystals^27–30^. For the present work we lump these contributions under the term amorphous, without further discussing their exact nature. The presence and intensity of reflections at 2*Θ* = 6.6°, 7.8°, and 9.0° corresponding to {020}, {120}, and {210} M1 crystal planes are used to assess the level of long-range ordering.

Although ordering in this mixed oxide series increased with higher V content, a thermal post-treatment in flowing N_2_ at 650 °C was necessary to fully form the M1 phase (Supplementary Fig. 3). Supplementary Tables 1 and 2 compile the characteristics of these thermally treated samples. The high-temperature post-treatment leads to crystallization of significant amounts (>30 wt. %) of inactive secondary phases. For these thermally treated samples the activity in ethane ODH increased with the concentration of V for a constant Te and Nb content (Supplementary Fig. 7). This agrees well with the established association of the catalytic activity with V^5+^ = O groups in M1 phase^9–12,31^.

Using a constant V/Mo ratio of 0.30 led, in contrast, to highly crystalline materials after hydrothermal synthesis at 190 °C, provided that the Te/Mo and Nb/Mo ratios were equal or lower than 0.10 (see Table 1, Supplementary Table 3 and Supplementary Fig. 4). This is the first observation of a direct hydrothermal formation of crystalline MoVTeNb-M1 phase. Using a lower V/Mo ratio (0.22) leads again to the formation of mixed oxides without long-range order, irrespective of the Nb and Te content (Supplementary Figs. 2 and 4). Again, these poorly crystalline materials can be transformed to M1 phase by treatment at 650 °C in inert atmosphere (Supplementary Figs. 3 and 5) but with formation of 10 to 40 wt.% of undesired crystalline phases.

**Table 1:** Properties of a series of MoVTeNbO_x_ materials prepared by the new method

Elemental composition^a^ (nominal formula^b^)	Surface area^c^/m² g^−1^	M1/wt.-%	Amorphous/wt.-%	Other phases
MoV_0.30_O_X_^d^ (MoV_0.30_O_X_)	63	n.d.^e^	n.d.^e^	n.d.^e^
MoV_0.30_Te_0.05_Nb_0.05_O_X_^d^ (MoV_0.30_Te_0.05_Nb_0.05_O_X_)	59	77	22	None
MoV_0.31_Te_0.05_Nb_0.10_O_X_^d^ (MoV_0.30_Te_0.05_Nb_0.10_O_X_)	71	58	40	NbO_2_
MoV_0.31_Te_0.12_Nb_0.08_O_X_^d^ (MoV_0.30_Te_0.10_Nb_0.05_O_X_)	74	52	47	Te^0^
MoV_0.30_Te_0.10_Nb_0.09_O_X_^d^ (MoV_0.30_Te_0.10_Nb_0.10_O_X_)	87	59	40	Te^0^
MoV_0.29_Te_0.14_Nb_0.17_O_X_^d^ (MoV_0.30_Te_0.18_Nb_0.18_O_X_)	111	n.d.^e^	n.d ^e^	n.d.^e^
MoV_0.22_Te_0.01_Nb_0.03_O_X_^d^ (MoV_0.22_Te_0.05_Nb_0.05_O_X_)	48	n.d.^e^	n.d.^e^	n.d.^e^

^a^Elemental analysis by inductively coupled plasma optical emission spectrometry (ICP-OES)

^b^Stoichiometry of metals subjected to hydrothermal synthesis

^c^Calculated according to Brunnauer-Emmett-Teller (BET) method. Samples were degasified at 250 °C prior N_2_ isotherm

^d^Samples were dried overnight in air at 80 °C

^e^Diffractograms (Supplementary Fig. 4) resemble poorly crystallized M1. Rietveld refinement of the diffractogram was hence not possible due to missing long-range order in the sample

**Fig. 2:**
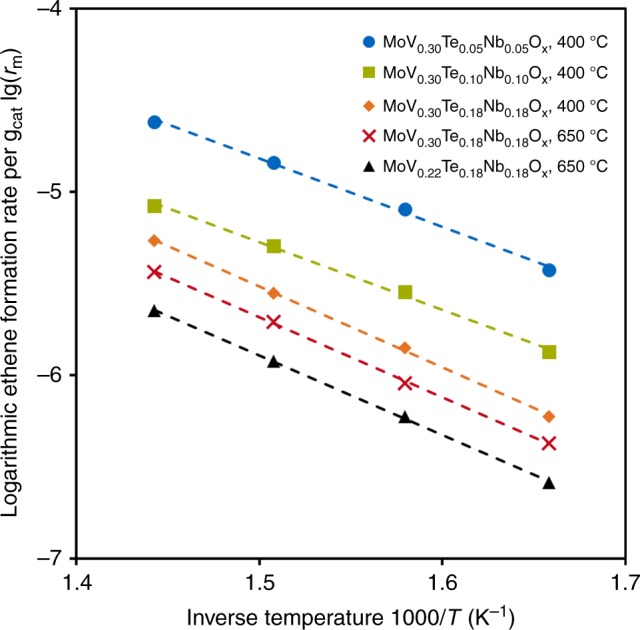
Arrhenius plot for ethene formation over selected MoVTeNbO_x_ samples. Temperatures of the different thermal treatments are given in the legend. Activity was normalized to catalyst mass. *T* = 330–420 °C, *p* = 1 bar (**a**), weight hourly space velocity (WHSV) = 14.3–17.6 h^−1^

The MoVTeNb oxides prepared by the new synthesis method with a stoichiometry in the range MoV_0.30_Te_0.05-0.10_Nb_0.05-0.10_O_x_ contain a significant fraction of the M1 phase (Table 1). Therefore, we studied the activity of selected samples from Supplementary Table 2 (high Te, Nb content) and Table 1 (low Te, Nb content) in a preconditioned state, i.e., treated in N_2_ at 400 °C for 2 h before reaction. The catalytic activities of two conventionally prepared samples serve as benchmark. Figure 2 shows that, even though ca. 50% of the solid was X-ray amorphous in the materials with low content in Te and Nb (Table 1), the activity per gram was significantly higher than for catalysts with high Te and Nb contents. In Fig. 2, the parallel lines indicate a constant apparent energy of activation (*E*_*A*_ = 81 ± 5 kJ mol^−1^). Thus, differences in the weight normalized activity among the different samples are attributed to differences in the pre-exponential factor, i.e., are caused by differences in the concentration of identical active sites.

### Origin of the high intrinsic activity

Having established the superior catalytic activity of the material in an optimal composition (i.e., MoV_0.30_Te_0.05_Nb_0.05_O_x_), we explore the role of crystallization temperature and thermal post-treatments on the final catalytic properties of M1 phases. Table 2 summarizes the physico-chemical properties of the MoV_0.30_Te_0.05_Nb_0.05_O_x_-mixed metal oxides prepared by the new method, after drying overnight at 80 °C and after being preconditioned in N_2_ to the ethane ODH reaction temperatures (400 °C). It should be noted that such preconditioning at 400 °C did not change the crystalline composition of the MoV_0.30_Te_0.05_Nb_0.05_O_x_ material as received from the hydrothermal step (see Supplementary Fig. 6). In Table 2 it is also shown the physico-chemical properties of catalysts with similar metal stoichiometry but prepared by a standard hydrothermal synthesis similar to the one described in ref. ^19^.

**Table 2:** Comparison of properties of M1 prepared by the new and standard methods

Elemental composition^a^ (nominal formula^b^)	Synthesis method	Thermal treatment	Surface area^c^/m² g^−1^	M1 phase content/wt. %	Crystallinity/wt. %
MoV_0.30_Te_0.05_Nb_0.05_O_x_ (MoV_0.30_Te_0.05_Nb_0.05_O_x_)	This work	80 °C, air	59	77	78
MoV_0.30_Te_0.05_Nb_0.05_O_x_ (MoV_0.30_Te_0.05_Nb_0.05_O_x_)	This work	400 °C, N_2_	50	79	82
MoV_0.31_Te_0.20_Nb_0.17_O_x_ (MoV_0.40_Te_0.10_Nb_0.10_O_x_)	Standard^19^	80 °C, air	61	n.d.^d^	n.d.^d^
MoV_0.30_Te_0.06_Nb_0.08_O_x_ (MoV_0.40_Te_0.10_Nb_0.10_O_x_)	Standard^19^	650 °C, N_2_	13	97	100

^a^Elemental analysis by inductively coupled plasma optical emission spectrometry (ICP-OES)

^b^Stoichiometry of metals subjected to hydrothermal synthesis

^c^Calculated according to Brunnauer-Emmett-Teller (BET) method. Samples were degasified at 250 °C prior N_2_ isotherm

^d^Diffractogram shows only a single broad reflection around 2*Θ* = 21.4° matching the {001}-plane reflections of e.g., M1, M2, and M_5_O_14_ phases. Rietveld refinement of the diffractogram was not possible due to missing long-range order in the sample

For the directly hydrothermally synthesized M1 catalyst, preconditioning the material at 400 °C in N_2_ flow resulted in a small loss of BET surface area and pore volume (Table 2 and Supplementary Table 4), and a slight increase in overall crystallinity and M1 phase content. Note that the specific surface area of the preconditioned mixed metal oxide was 50 m^2^ g^−1^. This is considerably larger than any specific surface areas previously reported for M1 phase Mo-V based catalysts^5,21,32,33^. It is also four times larger than the specific surface area of the reference sample.

**Fig. 3:**
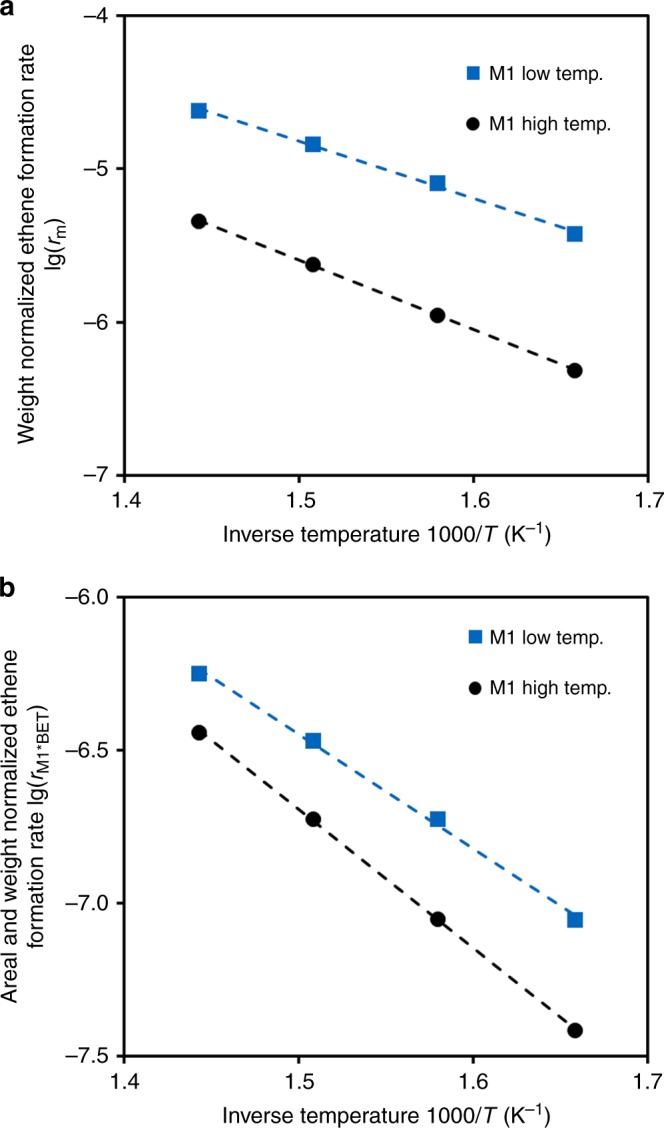
Arrhenius plot for ethene formation over M1 catalysts. The rate of a MoVTeNbOx catalyst prepared by the new synthesis method (low temp, MoV_0.30_Te_0.05_Nb_0.05_O_x_) is compared with the rate over M1 prepared by standard method as in ref. ^19^ (high temp, MoV_0.30_Te_0.06_Nb_0.08_O_x_). Rates normalized per mass of catalyst (**a**) and surface of M1 (**b**). *T* = 330–420 °C, *p* = 1 bar(**a**), WHSV = 6.8 h^−1^ (high temp sample), 14.5 h^−1^ (low temp sample)

Figure 3a shows the activity per gram in ODH-E of two M1 samples with similar metal stoichiometry prepared by the standard and the new synthesis methods. Normalization of the activity of the two MoVTeNbO_x_ catalysts of Table 2 to the weight of the M1 phase and the specific surface area (assuming that all phases contribute equally to the specific surface area) shows that the catalysts prepared via the new synthesis method have a higher intrinsic catalytic activity (Fig. 3b). Similar trends can be found for other normalization methods (Supplementary Fig. 8). It should be noted that a slightly lower selectivity towards ethene is observed for the sample prepared by the new synthesis method (Supplementary Fig. 9). Nevertheless, the ethene formation rate achieved by this high surface M1 catalyst is one order of magnitude higher than in a catalyst prepared by the standard method (Fig. 3a).

The apparent energy of activation for ethene formation was 75 ± 4 kJ mol^−1^ for the catalyst crystallized under hydrothermal conditions (190 °C) and 88 ± 1 kJ mol^−1^ for the reference catalyst crystallized at 650 °C. The 13 kJ mol^−1^ difference in *E*_*a*_ is not significant enough to be attributed to a change in the mechanism. Thus, for catalysts with a similar metal stoichiometry, it can be concluded that the M1 phase preparation route does not affect the main reaction pathways of ethane oxidation.

**Fig. 4:**
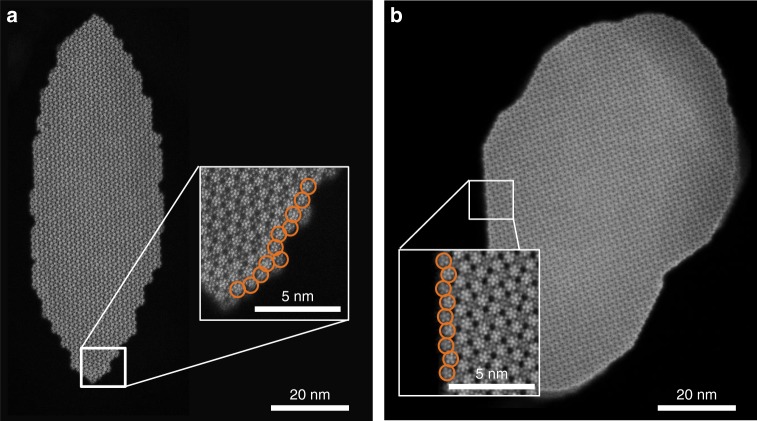
ADF-STEM images of {001} plane of M1 MoVTeNbO_x_ particles. Sample prepared by the new synthesis method, after drying overnight in air at 80 °C (**a**) and sample prepared by the method in ref. ^19^, followed by crystallization at 650 °C in inert (**b**). Insets show representative lateral surface termination. Circles highlight complete pentagonal M_6_O_21_ units observed near the termination

In order to understand the higher intrinsic activity, high-resolution STEM was used to determine the crystal termination of M1 particles (Fig. 4). The results show a corrugated lateral surface (Fig. 4a) for the sample prepared by the new method (thus, crystallized under hydrothermal conditions at 190 °C), while the particles prepared via the standard synthesis (thermally crystallized at 650 °C) present a smooth surface (Fig. 4b). Analysis of the particles showed that the smooth surfaces were {010} facets, formed by densely packed chains of pentagonal M_6_O_21_ building blocks (sites S5, S6, S8–S11 in Fig. 1) connected by single MO_6_ octahedra (sites S1 and S3). This facet exposes a very low surface concentration of active sites^16^. Materials with a lateral termination predominantly formed by {010} facets are expected to have low intrinsic activities. Conversely, M1 particles synthesized via the new method were terminated by a larger diversity of structural motifs, frequently including the S2-S4-S7 ensemble linked to the ODH-E activity of M1-MoVTeNbO_x_^7,10^. The corrugated lateral termination of low-temperature crystallized M1 particles is hypothesized to result from incomplete saturation of growth sites on the crystal surface; i.e., the side walls have not reached their thermodynamically most favored crystal termination, the lateral facet {010}.

Recently, it was hypothesized that sorption in heptagonal channels is responsible for the exceptional activity and selectivity of M1 in oxidation of small alkanes^13^. We cannot rule out that half-channel motifs on corrugated lateral surfaces could stabilize the transition state, causing the slightly lower apparent activation energy for ethene formation of the low-temperature synthesized samples.

### Mechanism of M1 crystallization in hydrothermal conditions

**Fig. 5:**
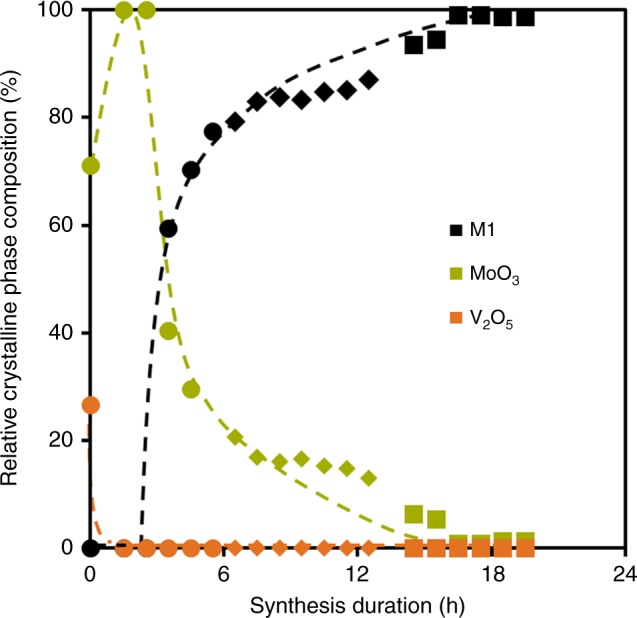
Relative crystalline phase composition of precipitate during synthesis. Solid fractions of aliquots were dried overnight in air at 80 °C prior XRD analysis. Different symbols are used for different synthesis batches. Initial mixture (*t* = 0 h) contains 4% of crystalline TeO_2_, omitted for the sake of clarity

In order to better understand the processes during hydrothermal synthesis, aliquots of the reacting slurry were periodically extracted. The crystalline composition of these mixtures as function of synthesis duration is shown in Fig. 5 (for details see Supplementary Table 5 and Supplementary Fig. 10). It should be noted that the small amount of solid collected did not allow quantification of the amorphous content. Therefore, percentages shown in Fig. 5 are given only with respect to the crystalline fraction. The evolution of crystalline phase composition of the solid fraction of these aliquots is comparable to the trend observed for MoVTeNbO_x_ materials prepared by the same method using short synthesis times (Supplementary Tables 5 and 6). The data point at 0 h corresponds to the composition of the physical mixture of the reactants. After 2.5 h, only MoO_3_ was observed. MoVTeNbO_x_-M1 was first detected after 3.5 h, but was at that point already the most abundant crystalline phase. Nearly all crystalline MoO_3_ were either dissolved or incorporated in M1-MoVTeNbO_x_ after 15 h. The crystalline composition of the mixture did not change further for hydrothermal synthesis times longer than 15 h.

**Fig. 6:**
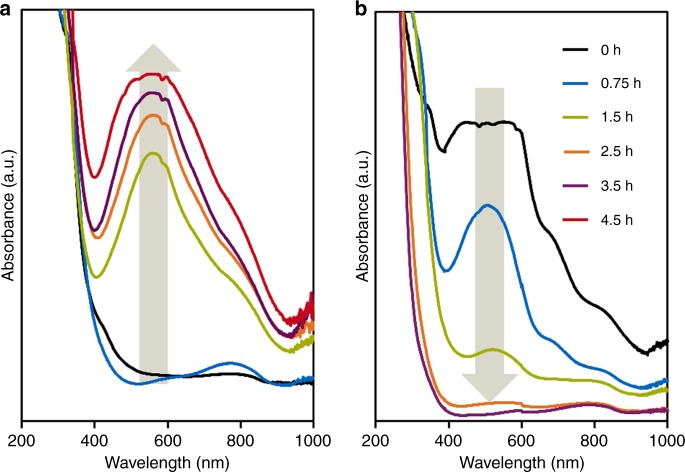
UV-Vis spectra of filtrates extracted from MoVTeNb hydrothermal slurries. Synthesis according to the procedure presented in this work in presence of additives (**a**); and synthesis according to procedure presented in ref. ^19^ (**b**)

Having established that the formation of the M1 phase occurs predominantly in the first 4.5 h, we focus on the speciation of the metal ions or clusters during this time interval. UV-vis absorption spectra of the filtrates (Fig. 6 and Supplementary Fig. 11) showed bands at 570, 670, and 800 nm for both synthesis routes. These bands are attributed to a {M_102_} ({Mo_72_V_30_}-type) Keplerate metal cluster, which contains 12 pentagonal M_6_O_21_ structural motifs connected by partially reduced metal-octahedra units^24,34–36^. This M_6_O_21_ motif is also found in M1, as seen in Fig. 1 (sites S5, S6, and S8–S11), and thus the ({Mo_72_V_30_}-type) Keplerate is regarded as an intermediate in the formation of M1-type metal oxides^19,24,25^.

In case of the synthesis method starting from metal oxides (Fig. 6a), the initial liquid phase does not show UV-vis absorption bands. With increasing synthesis time, first it is observed a band at ca. 800 nm that is tentatively assigned to V(IV) oxalate species in solution^37^. After 1.5 h, the absorption bands of {M_102_} appeared and their intensity increased with time. In contrast, for the standard recipe starting from soluble metal salts, the UV-vis spectra bands in Fig. 6b point to high concentrations of {M_102_} clusters in the initial solution, but rapidly decreasing over time until complete decomposition or precipitation at 2.5 h.

**Fig. 7:**
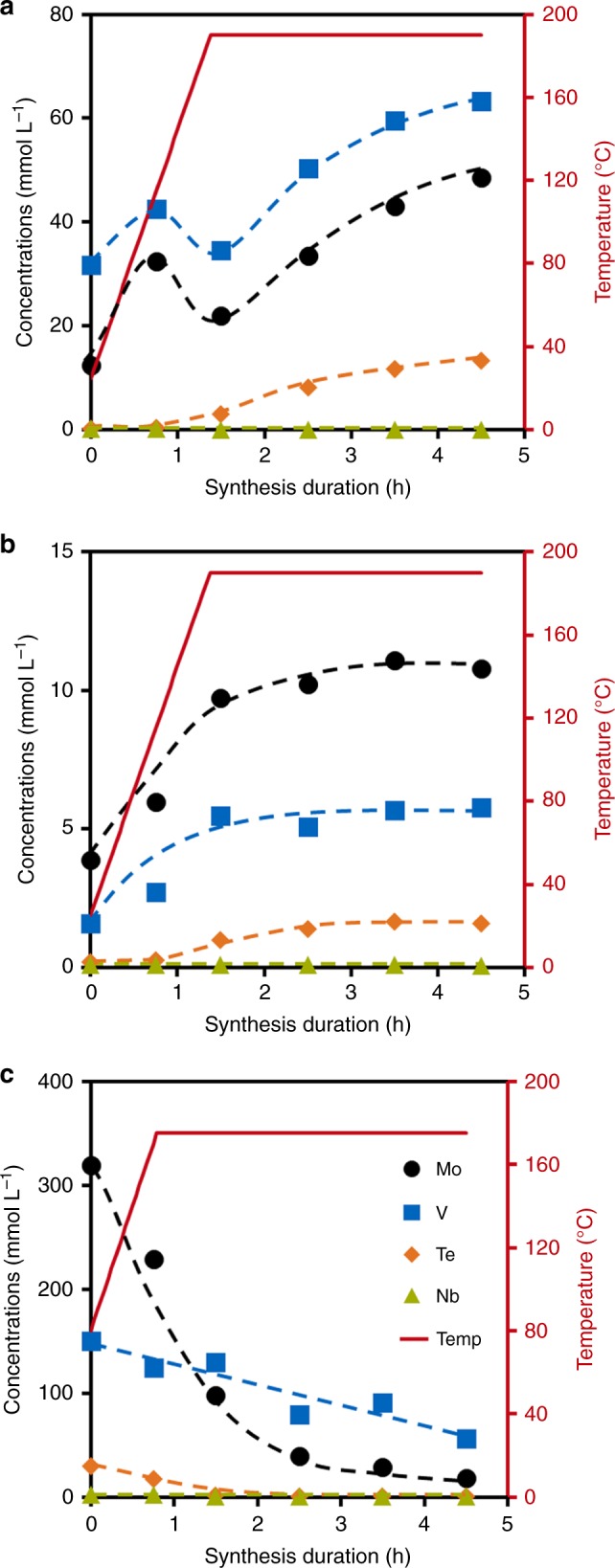
Composition of filtrates extracted from MoVTeNb hydrothermal slurries. Synthesis according to the procedure presented in this work in presence of additives (**a**); reference synthesis as in **a**, but without additives (**b**); synthesis according to procedure presented in^19^ **c**

Changes in the concentration of metal cations in the liquid phase within the first hours of hydrothermal synthesis are depicted in Fig. 7. Under hydrothermal conditions, the metal concentrations in solution in the new synthesis method increased with time (Fig. 7a). Comparison with Fig. 7b shows how the chelating agents citric acid, oxalic acid, and ethylene glycol aided the solubilization of metal ions from the oxide reactants. According to UV-vis spectra (Fig. 6a), once hydrothermal conditions have been reached after 1.5 h of synthesis, the main species in solution is a {Mo_72_V_30_} Keplerate cluster. If one assumes that at this point all Mo is forming {Mo_72_V_30_}, the concentration of this precursor increases from 0.3 to 0.7 mmol L^−1^ in the next hours, in the presence of a roughly constant excess of V (35–40 mmol L^−1^) and ~10 mmol L^−1^ of Te in solution (Supplementary Fig. 12).

When using metal salt reactants (Fig. 7c), initial concentrations of metal cations in solution were about two orders of magnitude larger than during the oxide-based method, but then decreased sharply during the first hours of hydrothermal synthesis. Again assuming that all Mo forms the Keplerate intermediate observed by UV-vis (Fig. 6b), the concentration of {Mo_72_V_30_} decreases from initial 4.5 mmol L^−1^ to only 0.5 mmol L^−1^ after 2.5 h, while the V excess in solution reaches ca 80 mmol L^−1^ at 1.5 h (Supplementary Fig. 13). This translates into an ionic strength approximately four times higher than the value estimated for the solution formed by oxides partially solubilized by complexing agents.

The formation of threshold concentrations of {Mo_72_V_30_} precursors in water has been found indispensable to form the M1 and other related MoVO_x_ phases^24,25,38,39^. We hypothesize, therefore, that maintaining the concentration of {Mo_72_V_30_} precursors just above this level sufficiently prevents uncontrolled precipitation of mixed oxides. We speculate that retarding the precipitation of {Mo_72_V_30_} allows time for the incorporation of V in linker positions between M_6_O_21_ units—and probably also the coordination of Te to V species—essential for the in situ crystallization of M1 phase. The subtlety of the approach is illustrated by the fact that the synthesis route did not lead to crystalline solids when higher concentrations of Nb (Nb/Mo > 0.10) were used. Niobium cations are known to strongly prefer the central position in M_6_O_21_ units^40^ and it is readily available as Nb^5+^ to form such motifs in the first instances of the hydrothermal synthesis. Thus, we hypothesize that the presence of Nb^5+^ accelerates the formation and precipitation of M_6_O_21_ units, and, for concentrations above 0.05 M (corresponding to nominal Nb/Mo > 0.10), the precipitation rate exceeds the M1 crystallization rate.

### Impact of M1 crystallization kinetics on ODH activity

As we have concluded that the crystal morphology is kinetically controlled, the crystallization time could conceptually have a significant impact on the concentration of active sites. A series of samples with nominal stoichiometry MoV_0.30_Te_0.05_Nb_0.05_O_x_ was, therefore, prepared via the method described in this work, allowing crystallization under hydrothermal conditions for 3.5–48 h (see Supplementary Table 6). Surprisingly, after only 3.5 h of synthesis, M1 phase constituted ~50% of the solid.

**Fig. 8:**
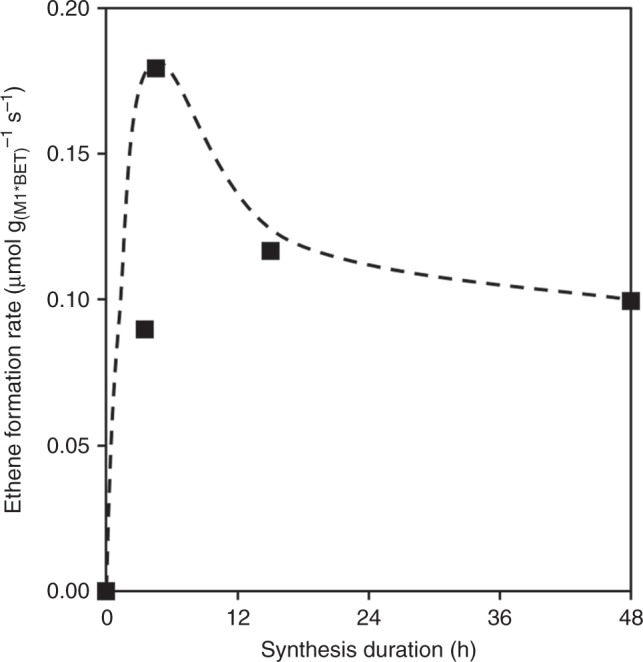
Ethene formation rates of MoV_0.30_Te_0.05_Nb_0.05_Ox synthesized at various times. Samples synthesized by the method described in this work. Reaction conditions *T* = 330 °C, *p* = 1 bar (**a**), WHSV = 7.0–13.8 h^−1^. Rates were normalized to M1 phase content and BET area

The activity in ethane ODH reached a maximum for synthesis durations of 4.5 h (Fig. 8 and Supplementary Fig. 14). Comparable energies of activation (73 ± 1 kJ mol^−1^) and selectivities as function of conversion (Supplementary Fig. 15) were observed for all samples, indicating an identical reaction mechanism on all catalysts. Thus, we attribute the reactivity differences to the variations in the concentrations of active sites. It should be also noted that normalization with respect to mass of M1 (as determined by XRD analysis) tends to overestimate the intrinsic activity for those samples containing active nanocrystalline M1, which, due to broad or absent diffraction lines, would be included in the amorphous mass during Rietveld analysis. The content in nanocrystalline M1 is expected to be significant in samples prepared at short synthesis times (≤4.5 h).

A size distribution of the length of the M1 needles was obtained by statistical analysis of SEM images of samples prepared at different synthesis durations (Supplementary Figs. 16 to 20). The length of M1 particles was affected only in early stages of hydrothermal synthesis, up to 4.5 h. For the sake of comparison, SEM images of a MoVTeNbOx material prepared by the standard method and crystallized at 650 °C were also taken (Supplementary Fig. 21). The M1 particles prepared in this way show a somewhat larger diameter/length aspect ratio and an overall shorter particle length (Supplementary Fig. 22).

STEM analysis showed that all materials prepared by the new synthesis method have a similar degree of surface corrugation of the M1 particles (Supplementary Figs. 23–26), although the number of particles analyzed for each sample does not allow for a quantification of surface density of active sites.

From the study of electron microscopy images, we conclude that synthesis time in the range 4.5–48 h does not affect significantly morphology and size of M1 particles. The special morphology and corrugation that translates into high activity in ODH is established in the first instants of the synthesis method (*t* < 3.5 h) and stem from the synthesis conditions that facilitate crystallization in the hydrothermal step. A certain decay of intrinsic activity of M1 for synthesis times above 4.5 h (Fig. 8 and Supplementary Fig. 14), is likely caused by subtle changes in the atomic surface termination of the particles. It is plausible that, with increasing synthesis time, deposition and reorganization of M_6_O_21_ and MO_6_ building blocks at high-energy facets become dominating, leading to less defective surfaces and thus to a decreasing concentration of active sites.

## Discussion

Highly active M1 type crystals MoVTeNbO_x_ have been directly and selectively synthesized under hydrothermal conditions using oxides with the aid of complexing agents that control the activities of ionic intermediates. This strategy maintains the concentrations of metal cations and in turn of polyoxometalate clusters at a level sufficiently high for crystallization and sufficiently low to avoid formation of amorphous mixed oxides.

The mixed metal oxide catalysts prepared by the synthesis path described in this work exhibit an unusually high intrinsic activity for ethane ODH. We attribute this superior performance to the formation of M1 crystals with highly corrugated side walls that expose a large concentration of active sites. The catalytic activity reaches a maximum after ca. 4 h under hydrothermal conditions, followed by a period in which surface restructuring and defects healing are hypothesized to lead to a slight decrease of activity.

The synthesis method described here offers the advantage of using inexpensive and abundant metal oxide reactants and the simplicity of one-batch synthesis, enabling straightforward scale-up of the synthesis.

The present study not only provides a pathway to highly active materials, it also confirms the proposed structures of the active sites and the sensitivity to small variations in local structures of the active site. It shows that catalyst design and synthesis can be realized from first principles.

## Methods

### MoVTeNbOx synthesis method starting from metal oxides

MoO_3_, V_2_O_5_, TeO_2_, Nb_2_O_5_∙1.5H_2_O, citric acid (CA), oxalic acid (OA), and monoethylene glycol (EG) are mixed as an aqueous dispersion (millipore grade)^41,42^. All reactants were used as purchased without further purification. The concentration of Mo was fixed at 0.5 mol L^−1^. Metal stoichiometry was varied in the range of Mo:V:Te:Nb = 1:0.22–0.30:0–0.18:0–0.18. Concentration of synthesis additives relative to Mo was varied in the range Mo:CA:OA:EG = 1:0.075:0–0.18:0.075. The ratio Nb:OA was fixed at 1:1. Hydrothermal synthesis was performed in two different types of autoclaves: (A) Teflon lined stainless steel autoclaves without controlled stirring, but attached to a rotating shaft, and heated in a muffle furnace (volume H_2_O = 75 mL), were used for the study of synthesis parameters, and (B) a Premex Reactor AG hpm-p stainless steel autoclave including a blade stirrer (volume H_2_O = 200 mL) heated by an external thermostat (Lauda E4G) was used for study of hydrothermal crystallization kinetics. All syntheses were performed at 190 °C and 17.5 bar(a) for 48 h except otherwise stated in the manuscript. Once the temperature program has finished, the reacting slurry in the vessel is cooled down from reaction temperature to <30 °C in about 1.5 h while maintaining stirring. The solid is then separated from the synthesis liquor by filtration (2–3 µm), washed with 2.5 times the volume of ultra-pure water used in the initial reaction mixture and dried in static air at 80 °C.

In some cases, aliquots (about 5 mL, <10 mg solid) have been extracted from the autoclave and immediately quenched in order to analyze the evolution of the slurry with time during hydrothermal synthesis. The solid was separated from the liquor by filtration, washed with ca. 12 mL of Millipore grade water and dried at 80 °C in static air.

As an example, we give here the details for synthesis of the mixed oxide with the nominal composition MoV_0.30_Te_0.05_Nb_0.05_O_x_ in the 300 mL Premex autoclave. In the first step, 5397 mg MoO_3_, 1023 mg V_2_O_5_, 299 mg TeO_2_, 275 mg Nb_2_O_5_∙1.5H_2_O, 540 mg citric acid, 169 mg oxalic acid, and 175 mg ethylene glycol are dispersed simultaneously in 200 mL of Millipore grade water at ambient temperature. The autoclave was immediately sealed without purging the remaining air volume with inert gas. The stirring of the mixture at ca. 750 rpm started simultaneously with temperature ramp.

### Reference MoVTeNbOx synthesis starting from metal salts

5.33 mmol (NH_4_)_6_Mo_7_O_24_∙4H_2_O are dissolved in 30 mL of H_2_O (Millipore grade) at 80 °C^19^. Aqueous solutions of 14.92 mmol VOSO_4_, 3.73 mmol Te(OH)_6_, and 3.73 mmol (NH_4_)NbO(C_2_O_4_)_2_ (80 °C and 15 mL, each) are added stepwise to the ammonium heptamolybdate solution. The mixture is vigorously stirred for 10 min before being transferred to a teflon lined stainless steel autoclave (Autoclave A). Hydrothermal synthesis was performed at 175 °C and autogenous pressure for 20 h. The precipitates were separated from the synthesis liquor by filtration (2–3 µm) and washed with 2.5 times the volume of ultra-pure water used in the initial reaction mixture. For synthesis in autoclave B, amounts of metal reactants and H_2_O are scaled to a liquid volume of 200 mL.

### Thermal treatment

All crude precipitated materials were dried in static air at 80 °C for 16 h after filtration (materials denoted as as-synthesized or low-temperature crystallized). Optionally, two different thermal treatments could be applied to dried Mo-V-Te-Nb mixed metal oxide. (A) Preconditioning to reaction temperature at 400 °C in flowing N_2_ with a heating ramp of 15 °C min^−1^ and dwell time of 2 h (materials denoted as preconditioned). (B) Two-step profile with heating to 200 °C at 15 °C min^−1^ in flow of synthetic air and 2 h dwell time followed by 30 min flushing with flow of N_2_ and subsequent heating to 650 °C at 15 °C min^−1^ in flow of N_2_ and dwell time of 2 h (materials denoted as high-temperature crystallized).

### X-ray diffraction analysis

All measurements of the powdered samples were performed on a PANalytical Empyrian or PANalytical X’pert Pro diffractometers built in a Bragg-Brentano geometry (θ -2θ-goniometer), using copper-Kα radiation and operating at 45 kV and 40 mA. The scanning range was 5° to 70° 2θ with increments of 0.017°. For the quantification of the amorphous content, the metal oxide sample was thoroughly mixed with about 10 wt.-% of a fully crystalline Rutile (r-TiO_2_) standard material obtained from NIST. Diffractogram and Rietveld analysis was performed using PANalytical Highscore Plus v3 software. Quantification of the amorphous content was done according to the procedure described elsewhere^16^.

### Surface area, pore volume, and pore size distribution

N_2_ physisorption was carried out at −196 °C using a Sorptomatic 1990 automated surface area and pore size analyzer. Prior to the measurements all samples were evacuated at a temperature of 250 °C and a pressure of 10^−2^ mbar for 2 h. Surface area was calculated according to the Brunnauer-Emmett-Teller (BET) method and pore volumes and pore size distribution were calculated using the Barrett-Joyner-Halenda (BJH) model. Error of the measurement is <1%.

### Elemental analysis

Elemental analysis was performed on an Agilent 760 ICP-OES spectrometer. Metal concentrations were determined using 281.615 nm (Mo), 311.817 nm (V), 214.282 nm (Te), and 3131.078 nm (Nb) emission lines. Solid mixed metal oxide samples were fused in soda-potash mixture before being dissolved in millipore grade water.

### UV-vis

UV-vis spectra were recorded in absorbance mode on a Hitachi U3000 spectrometer in a wavelength range of 200–1000 nm with a scanning rate of 600 nm min^−1^ using a slit with of 5 nm in a commercial quartz cuvette with Millipore grade water in an identical cuvette as a reference.

### Scanning electron microscopy

The morphology of M1 phase particles was characterized using a high-resolution scanning electron microscope 7500 F ColdFEG (JEOL) operating at an accelerating voltage of 2.0 kV and emission current of 10 µA. Working distance was about 8 mm.

### Scanning transmission electron microscopy

Characterization of the edge termination of synthesized M1 crystals was carried out on a Thermo Fisher Scientific aberration-corrected Titan 80/300 TEM/STEM operated at 300 keV, housed in the Environmental Molecular Sciences Laboratory (EMSL) at the Pacific Northwest National Laboratory (PNNL). Correcting the spherical aberration by the illumination aberration corrector, Titan STEM allows a larger beam convergence angle, providing a small electron probe of high current. All high-angle annular dark-field (HAADF) STEM micrographs reported here were acquired with a convergence semi-angle of 17.4 mrad after fine tuning of the probe corrector, and an ADF detector semi-angles of around 70–200 mrad. Special care was taking to limit electron dose and prevent electron-beam effects on the catalysts. TEM samples of the M1 catalysts were prepared by standard microtome technique.

### Catalytic activity

The activity of the catalyst samples was tested in a fixed bed plug flow reactor setup at atmospheric pressure in the temperature range of 330–420 °C. Up to 100 mg (depending on the activity of the sample) of catalyst (150–212 µm) were diluted in with SiC (150–212 µm) in the ratio of 1:5 in order to achieve homogeneous heat distribution within the catalyst bed. The gas feed flow was 50 sccm and composed of 9.1 mol-% ethane, 9.1 mol-% oxygen, and 81.8 mol-% helium. Weight hourly space velocity was adjusted to a maximum ethane conversion of *X*(C_2_H_6_) < 15 % in Arrhenius type experiments in order to suppress secondary reaction pathways. Online gas analysis was performed using a Shimadzu GC 2014 gas chromatography system capable of quantifying C_2_-hydrocarbons and -oxygenates, carbon mono- and dioxide, oxygen, and nitrogen. The main reaction product was ethene under all tested conditions. Carbon monoxide, carbon dioxide, and traces of acetic acid were the only detected side products.

## Supplementary information

Supplementary Information

Source Data

## Data Availability

The source data underlying for Figs. 2, 3, 5–8 are provided as a Source Data file. All other data supporting the findings in this study are available from the authors on request.
