# Supplementary material for: Design and synthesis of highly active MoVTeNb-oxides for ethane oxidative dehydrogenation
Source: Nat Commun. 2019 Sep 5;10:4012. doi: 10.1038/s41467-019-11940-0 (PMC6728333; doi:10.1038/s41467-019-11940-0)
Supplement: Supplementary file 1 — Supplementary Information [file 41467_2019_11940_MOESM1_ESM.pdf]

Design and synthesis of highly active MoVTeNb-oxides for ethane oxidative dehydrogenation

Daniel Melzer[1], Gerhard Mestl[2], Klaus Wanninger[2], Yuanyuan Zhu[3], Nigel D. Browning[4,5], Maricruz Sanchez-Sanchez[1]*, Johannes A. Lercher[1,4]*

[1] Department Chemie & Catalysis Research Center, TU München, Lichtenbergstr. 4, D-85747 Garching, Germany;

[2] Clariant Produkte (Deutschland) GmbH, Waldheimer Str. 13, D-83502 Bruckmühl, Germany

[3] Department of Materials Science and Engineering, Institute of Materials Science, University of Connecticut, Storrs, CT 06269, USA

[4] Institute for Integrated Catalysis, Pacific Northwest National Laboratory, Richland, WA 99352, USA

[5] Imaging Center at Liverpool (ICaL), School of Engineering & School of Physical Sciences, University of Liverpool. 506 Brodie Tower, Liverpool, L69 3GQ. UK

** Corresponding authors: m.sanchez@tum.de, johannes.lercher@ch.tum.de*

Supplementary Information

Supplementary Notes 1. Physico-chemical and structural characterization of different MoVTeNbOx samples

This section compiles the results from X-ray diffraction, nitrogen sorption and elemental analyses for all samples. For experimental details refer to the Methods section.

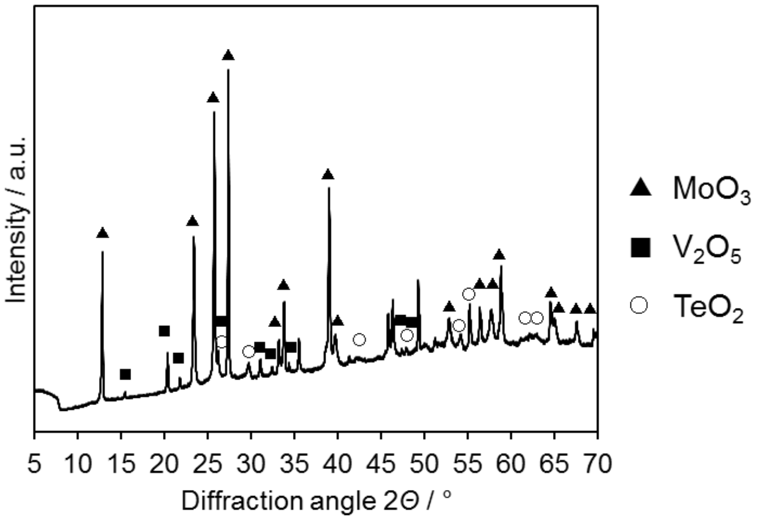

Supplementary Figure 1. Diffractogram of $MoV_{0.30}Te_{0.05}Nb_{0.05}O_x$ synthesized for 15 h at 190 °C by the new synthesis method but in absence of synthesis additives. The sample was dried in air overnight at 80 °C.

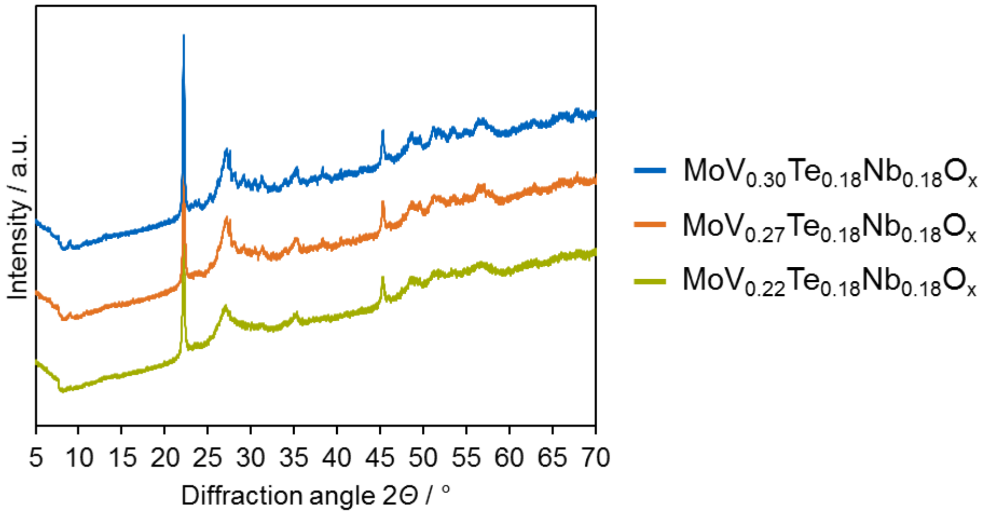

Supplementary Figure 2. Diffractogram of $MoV_{0.22-0.30}Te_{0.18}Nb_{0.18}O_x$ materials prepared by the new synthesis method, after drying overnight in air at 80 °C.

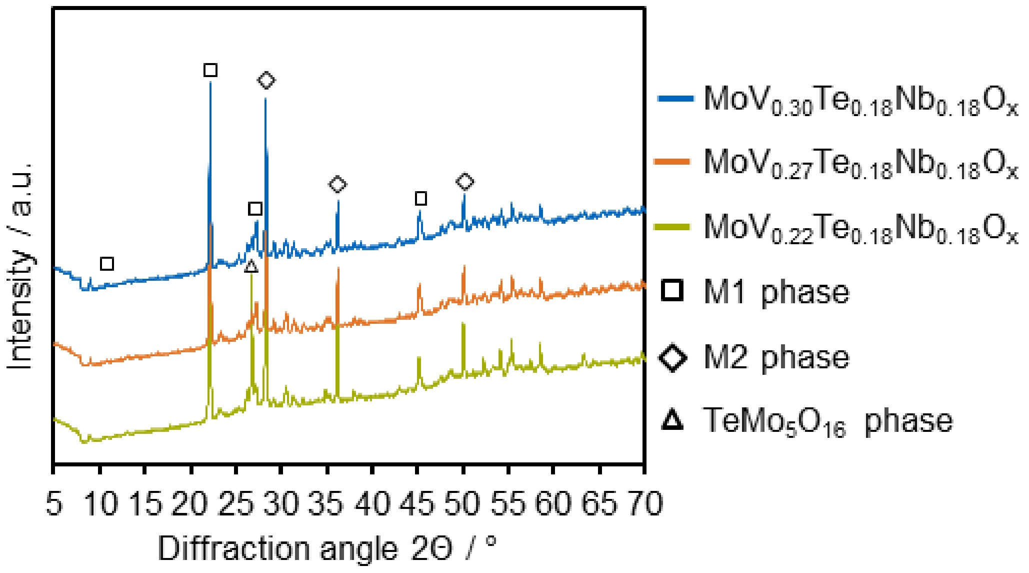

Supplementary Figure 3. Diffractograms of $MoV_{0.22\text{-}0.30}Te_{0.18}Nb_{0.18}O_x$ catalysts shown in Supplementary Figure 2 after thermal treatment at 650 °C. Labels mark main reflections of constituent crystal phases.

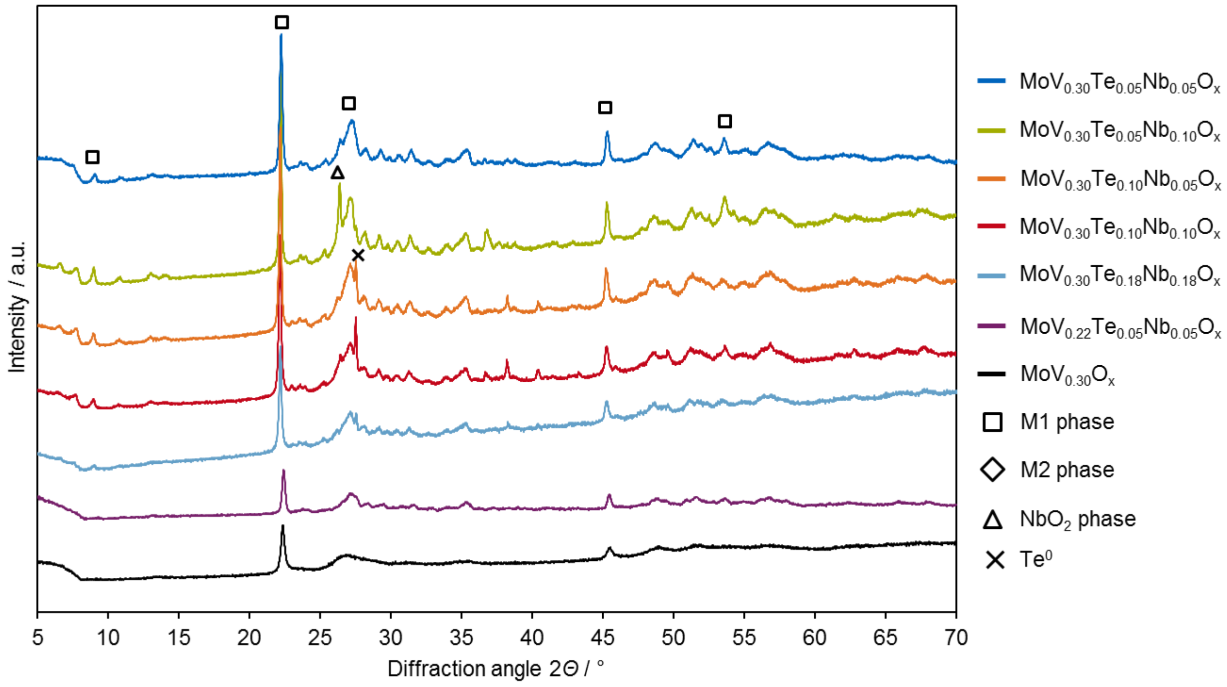

Supplementary Figure 4. Diffractograms of $MoVTeNbO_x$, synthesized via the new synthesis method, after drying overnight in air at 80 °C. Labels mark main reflections of crystal phases present above trace level.

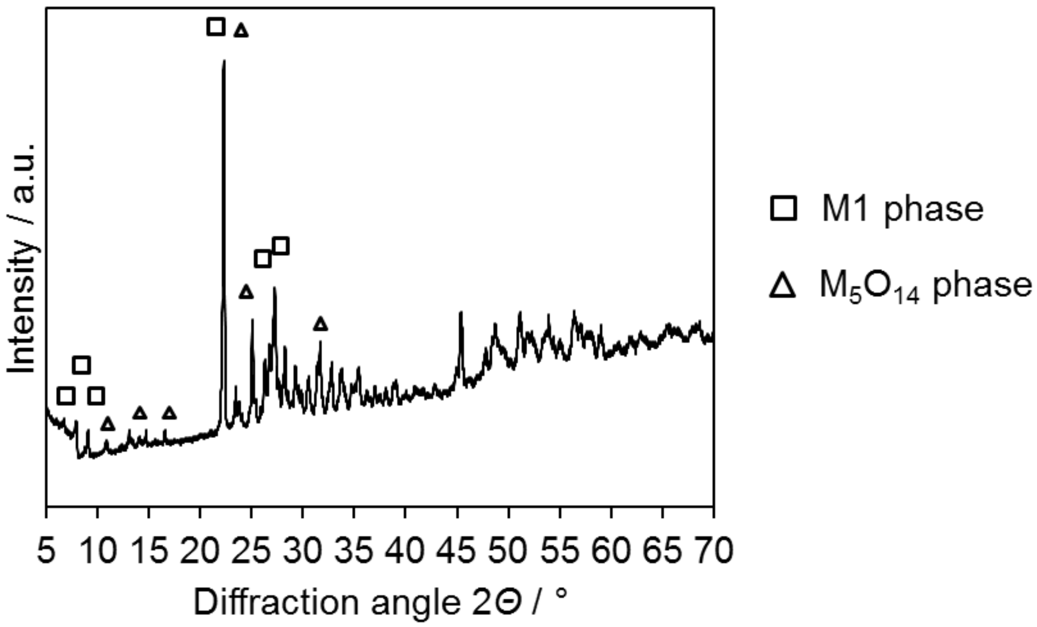

Supplementary Figure 5. Diffractogram of $MoV_{0.22}Te_{0.05}Nb_{0.05}O_x$, shown in Figure S4, after thermal treatment at 650°C.

Supplementary Table 1. Results of Rietveld refinement of all tested samples. Mass fractions of phases normalized to totality of material. For sample identification refer to main text.

ICP-OES (in brackets: nominal) formula	Thermal treatment	M1 / wt.-%	M2 / wt.-%	M_5O_{14} / wt.-%	MoO_3 / wt.-%	Amorphous / wt.-%	GoF / -
$MoV_{0.21}Te_{0.08}Nb_{0.17}O_x$ ($MoV_{0.22}Te_{0.18}Nb_{0.18}O_x$)	650 °C, N_2	61	39	0	0	0	2.8
$MoV_{0.25}Te_{0.07}Nb_{0.16}O_x$ ($MoV_{0.27}Te_{0.18}Nb_{0.18}O_x$)	650 °C, N_2	64	36	0	0	0	3.1
$MoV_{0.29}Te_{0.11}Nb_{0.18}O_x$ ($MoV_{0.30}Te_{0.18}Nb_{0.18}O_x$)	650 °C, N_2	58	33	0	0	0	2.9
$MoV_{0.30}O_x$ ($MoV_{0.30}O_x$)	80 °C, air	n.d. a)	n.d. a)	n.d. a)	n.d. a)	n.d. a)	n.d. a)
$MoV_{0.30}Te_{0.05}Nb_{0.05}O_x$ ($MoV_{0.30}Te_{0.05}Nb_{0.05}O_x$)	80 °C, air	77	0	0	1	22	21.4
$MoV_{0.31}Te_{0.05}Nb_{0.10}O_x$ ($MoV_{0.30}Te_{0.05}Nb_{0.10}O_x$)	80 °C, air	58	2	0	0	40	29.5
$MoV_{0.31}Te_{0.12}Nb_{0.08}O_x$ ($MoV_{0.30}Te_{0.10}Nb_{0.05}O_x$)	80 °C, air	52	1	0	0	47	20.4
$MoV_{0.30}Te_{0.10}Nb_{0.09}O_x$ ($MoV_{0.30}Te_{0.10}Nb_{0.10}O_x$)	80 °C, air	59	1	0	0	40	12.7
$MoV_{0.29}Te_{0.14}Nb_{0.17}O_x$ ($MoV_{0.30}Te_{0.18}Nb_{0.18}O_x$)	80 °C, air	n.d. a)	n.d. a)	n.d. a)	n.d. a)	n.d. a)	n.d. a)
$MoV_{0.22}Te_{0.01}Nb_{0.03}O_x$ ($MoV_{0.22}Te_{0.05}Nb_{0.05}O_x$)	80 °C, air	n.d. a)	n.d. a)	n.d. a)	n.d. a)	n.d. a)	n.d. a)
$MoV_{0.30}Te_{0.05}Nb_{0.05}O_x$ ($MoV_{0.30}Te_{0.05}Nb_{0.05}O_x$)	400 °C, N_2	79	0	0	3	18	5.8
$MoV_{0.31}Te_{0.20}Nb_{0.17}O_x$ ($MoV_{0.40}Te_{0.10}Nb_{0.10}O_x$)	80 °C, air	n.d. a)	n.d. a)	n.d. a)	n.d. a)	n.d. a)	n.d. a)
$MoV_{0.30}Te_{0.06}Nb_{0.08}O_x$ ($MoV_{0.40}Te_{0.10}Nb_{0.10}O_x$)	650 °C, N_2	97	0	0	0	3	15.4

a) Rietveld refinement of the diffractogram was not possible due to missing long range order in the sample.

Supplementary Table 2. Physico-chemical properties of $MoV_{0.22-0.30}Te_{0.18}Nb_{0.18}O_x$ catalysts after thermal treatment at 650°C.

ICP-OES (in brackets: nominal[a]) formula	BET / $m^2 g^{-1}$	M1 content / wt.-%	Amorphous content / wt.-%	Other phases
$MoV_{0.21}Te_{0.08}Nb_{0.17}O_x$ ($MoV_{0.22}Te_{0.18}Nb_{0.18}O_x$)	11	61	0	M2
$MoV_{0.25}Te_{0.07}Nb_{0.16}O_x$ ($MoV_{0.27}Te_{0.18}Nb_{0.18}O_x$)	22	64	0	M2
$MoV_{0.29}Te_{0.11}Nb_{0.18}O_x$ ($MoV_{0.30}Te_{0.18}Nb_{0.18}O_x$)	30	58	9	M2

a) Stoichiometry of metals subjected to hydrothermal synthesis

Supplementary Table 3. Nitrogen adsorption data of MoVTeNbOx materials prepared by to new synthesis method, after drying overnight in air at 80 °C.

ICP-OES (in brackets: nominal[a]) formula	BET[b] / $m^2 g^{-1}$	Internal area / $m^2 g^{-1}$	Pore volume / $cm^3 g^{-1}$
$MoV_{0.30}Te_0Nb_0O_X$ ($MoV_{0.30}Te_0Nb_0O_X$)	97	30	0.11
$MoV_{0.30}Te_{0.05}Nb_{0.05}O_X$ ($MoV_{0.30}Te_{0.05}Nb_{0.05}O_X$)	63	37	0.11
$MoV_{0.31}Te_{0.05}Nb_{0.10}O_X$ ($MoV_{0.30}Te_{0.05}Nb_{0.10}O_X$)	71	93	0.16
$MoV_{0.31}Te_{0.12}Nb_{0.08}O_X$ ($MoV_{0.30}Te_{0.10}Nb_{0.05}O_X$)	74	9	0.03
$MoV_{0.31}Te_{0.10}Nb_{0.09}O_X$ ($MoV_{0.30}Te_{0.10}Nb_{0.10}O_X$)	87	49	0.19
$MoV_{0.29}Te_{0.14}Nb_{0.17}O_X$ ($MoV_{0.30}Te_{0.18}Nb_{0.18}O_X$)	111	77	0.27
$MoV_{0.22}Te_{0.01}Nb_{0.03}O_X$ ($MoV_{0.22}Te_{0.05}Nb_{0.05}O_X$)	48	33	0.09

a) Stoichiometry of metals subjected to hydrothermal synthesis

b) Samples were degasified at 250 °C prior N_2 isotherm (see methods)

Supplementary Table 4. Nitrogen adsorption data of MoVTeNbOx materials prepared according to the new hydrothermal method described in this work and of MoVTeNbOx materials prepared by the standard hydrothermal synthesis method in [1].

ICP-OES (in brackets: nominal[a]) formula	Synthesis method	Thermal treatment	BET[b] / $m^2 g^{-1}$	Internal area / $m^2 g^{-1}$	Pore volume / $cm^3 g^{-1}$
$MoV_{0.30}Te_{0.05}Nb_{0.05}O_x$ ($MoV_{0.30}Te_{0.05}Nb_{0.05}O_x$)	This work	80 °C, air	59	30	0.11
$MoV_{0.30}Te_{0.05}Nb_{0.05}O_x$ ($MoV_{0.30}Te_{0.05}Nb_{0.05}O_x$)	This work	400 °C, N_2	50	25	0.09
$MoV_{0.31}Te_{0.20}Nb_{0.17}O_x$ ($MoV_{0.40}Te_{0.10}Nb_{0.10}O_x$)	Standard[1]	80 °C, air	61	93	0.12
$MoV_{0.30}Te_{0.06}Nb_{0.08}O_x$ ($MoV_{0.40}Te_{0.10}Nb_{0.10}O_x$)	Standard[1]	650 °C, N_2	13	9	0.03

a) Stoichiometry of metals subjected to hydrothermal synthesis

b) Samples were degasified at 250 °C prior N_2 isotherm (see methods)

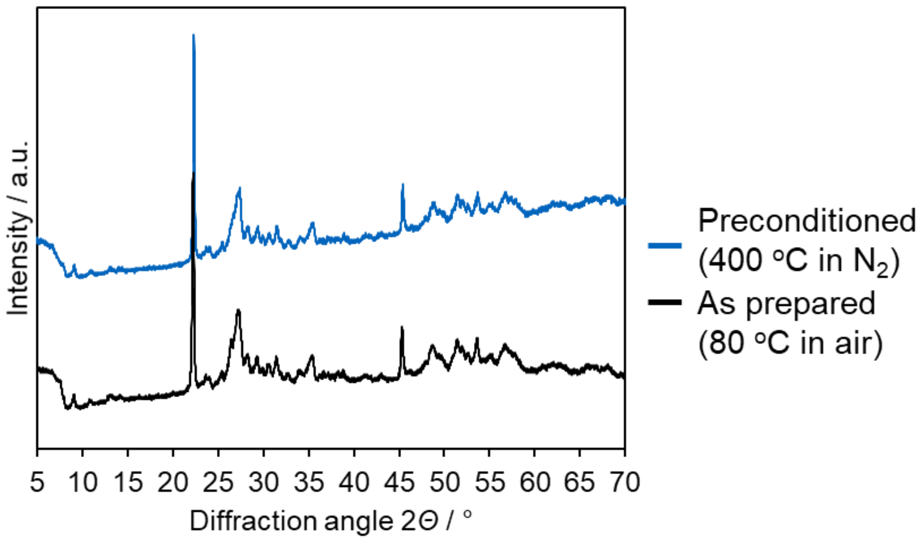

Supplementary Figure 6. Diffractograms of $MoV_{0.30}Te_{0.05}Nb_{0.05}O_x$ material as prepared (dried overnight at 80 °C in air) and preconditioned (after thermal treatment at 400°C in N_2). Results of Rietveld analysis are provided in Table 2 of main manuscript.

Supplementary Notes 2. Additional activity data of new and reference MoVTeNbOx syntheses

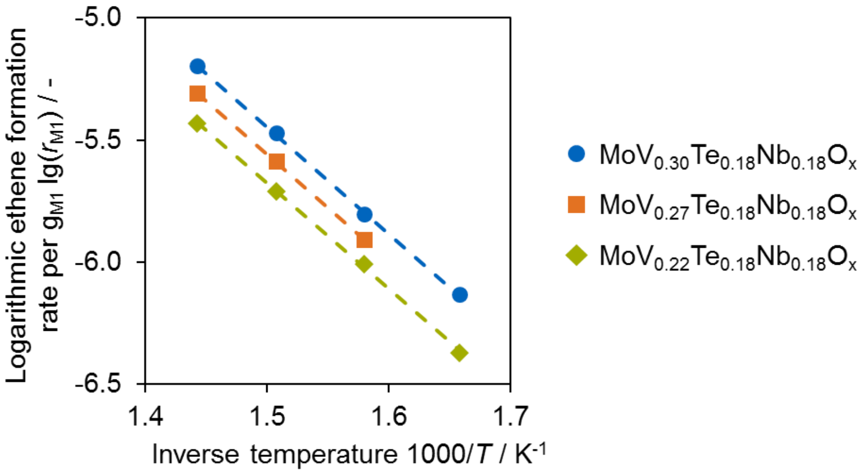

Supplementary Figure 7. Activity normalized to M1 phase content of samples containing different concentrations of vanadium and thermally treated at 650°C. Reaction conditions of ethane ODH: T = 330 – 420 °C, p = 1 bar(a), WHSV = 17.5 h^{-1}.

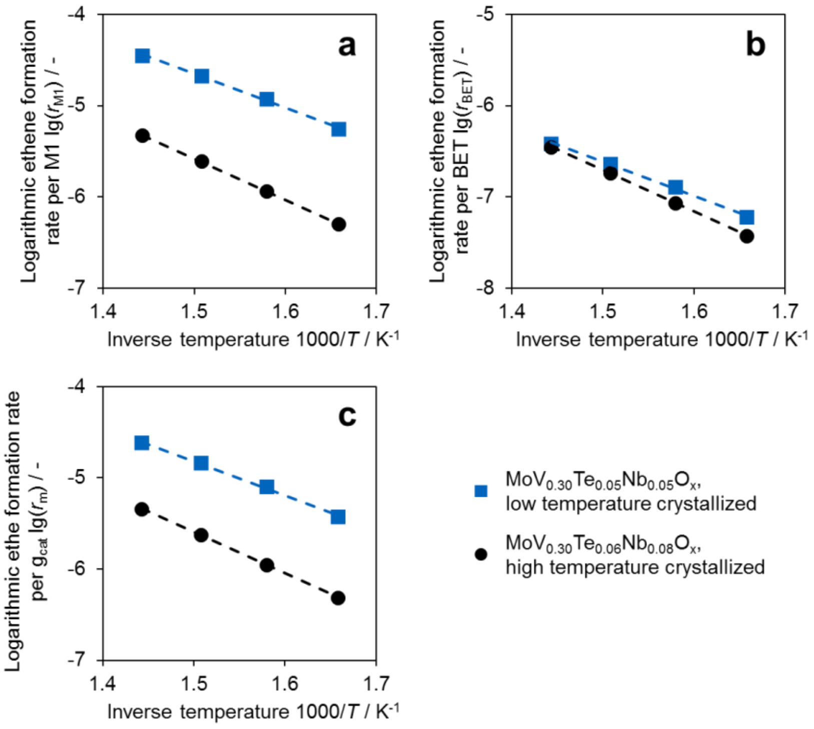

Supplementary Figure 8. Arrhenius type plot of a MoVTeNbOx synthesized by the new method (low temperature crystallization, blue squares) and MoVTeNbOx synthesized by the standard method (high temperature, black circles). Ethene formation rates normalized to M1 content (a), specific surface area (b) and mass mixed oxide sample (c).

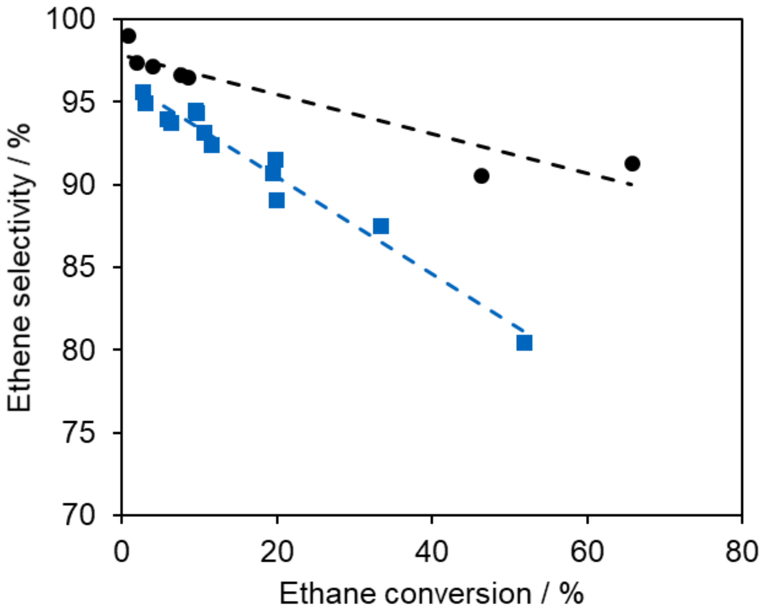

Supplementary Figure 9. Selectivity to ethene as function of ethane conversion obtained for a a MoVTeNbOx synthesized by the new method (low temperature crystallization, blue squares) and MoVTeNbOx synthesized by the standard method (high temperature, black circles).

Supplementary Notes 3. Effect of hydrothermal synthesis duration on catalytic activity

XRD and Rietveld analysis of MoVTeNbOx materials

Supplementary Table 5. Results of Rietveld refinement of solid aliquots extracted from hydrothermal synthesis of $MoV_{0.30}Te_{0.05}Nb_{0.05}O_x$ at different times, after drying overnight in air at 80 °C. Mass fractions of phases are given with respect to crystalline part of the material.

Synthesis time of aliquot / h	M1 / wt.-%	MoO_3/ wt.-%	V_2O_5/ wt.-%	TeO_2/ wt.-%	GoF / -
0	0	71	27	2	n.d.
1.5	0	100	0	0	3.3
2.5	0	100	0	0	4.3
3.5	60	41	0	0	4.8
4.5	70	30	0	0	4.2
5.5	78	23	0	0	4.1
6.5	79	21	0	0	4.2
7.5	83	17	0	0	3.9
8.5	84	16	0	0	3.7
9.5	83	17	0	0	3.9
10.5	85	15	0	0	4.0
11.5	85	15	0	0	3.6
12.5	87	13	0	0	3.8
14.5	94	6	0	0	4.9
15.5	95	6	0	0	4.36
16.5	99	1	0	0	3.7
17.5	99	1	0	0	3.7
18.5	99	1	0	0	4.2
19.5	99	1	0	0	4.1

Supplementary Table 6. Results of Rietveld refinement of metal oxide reactants, physical mixture of metal oxides with nominal stoichiometry Mo:V:Te:Nb = 1:0.30:0.05:0.05 and of $MoV_{0.30}Te_{0.05}Nb_{0.05}O_x$ materials prepared by the new synthesis method for different durations of the hydrothermal step. Samples were dried overnight in air at 80 °C after hydrothermal synthesis. Mass fractions of phases with respect to totality of material.

Sample	BET / $m^2 g^{-1}$	MoO_3 / wt.-%	V_2O_5 / wt.-%	TeO_2 / wt.-%	Nb_2O_5 / wt.-%	M1 / wt.-%	Amorphous / wt.-%	GoF / -
MoO_3		41	0	0	0	0	59	242.2
V_2O_5		0	100	0	0	0	0	71.1
TeO_2		0	0	32	0	0	68	206.3
Nb_2O_5•$1.5H_2O$		n.d. [a)]	n.d. [a)]	n.d. [a)]	n.d. [a)]	n.d. [a)]	n.d. [a)]	n.d. [a)]
Phys. mix. / 0 h		34	13	1	0	0	52	63.9
3.5 h	67	14	0	0	0	48	38	27.1
4.5 h	73	17	0	0	0	55	29	26.9
15 h	68	5	0	0	0	76	19	28.2
48 h	40	1	0	0	0	77	22	9.2

a) Rietveld refinement of the diffractogram was not possible due to missing long range order in the sample.

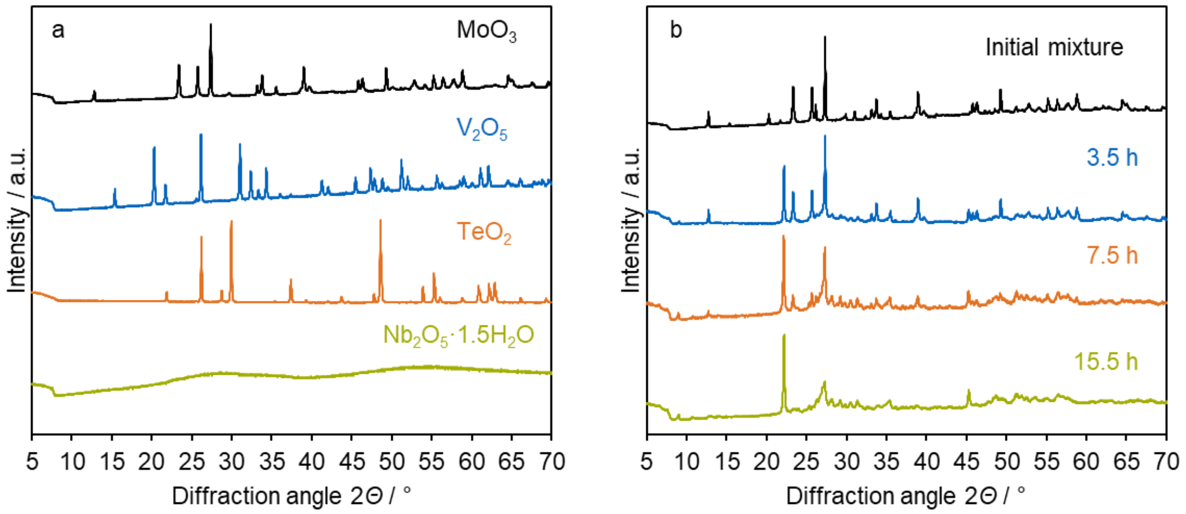

Supplementary Figure 10. X-ray diffractograms of reactants (a) and selected aliquots obtained during hydrothermal synthesis (190 °C, 17.5 bar(a)) of $MoV_{0.30}Te_{0.05}Nb_{0.05}O_x$ according to new method (b). Samples were dried overnight at 80 °C in air.

UV-vis absorption spectroscopy

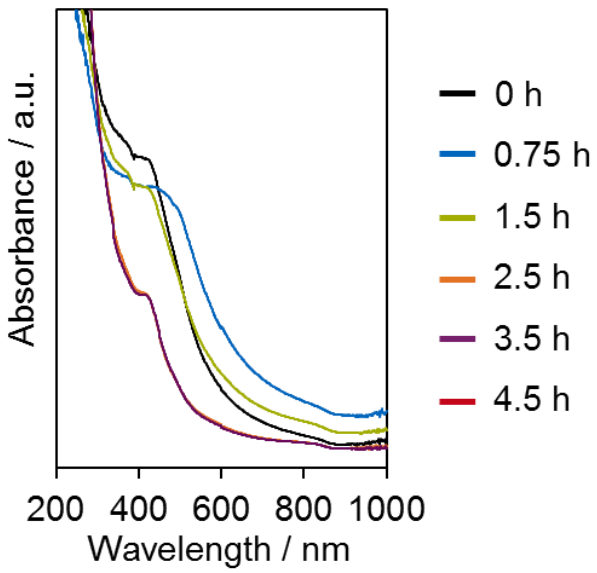

Supplementary Figure 11. UV-Vis spectra of filtrates extracted from MoVTeNb slurry at various times during hydrothermal synthesis in absence of additives.

Concentrations of Mo and V species in solution

Supplementary Figures 12 and 13 show calculated concentrations of {M_{102}} kelplerate and excess V species in aqueous solution during first hours of hydrothermal synthesis.

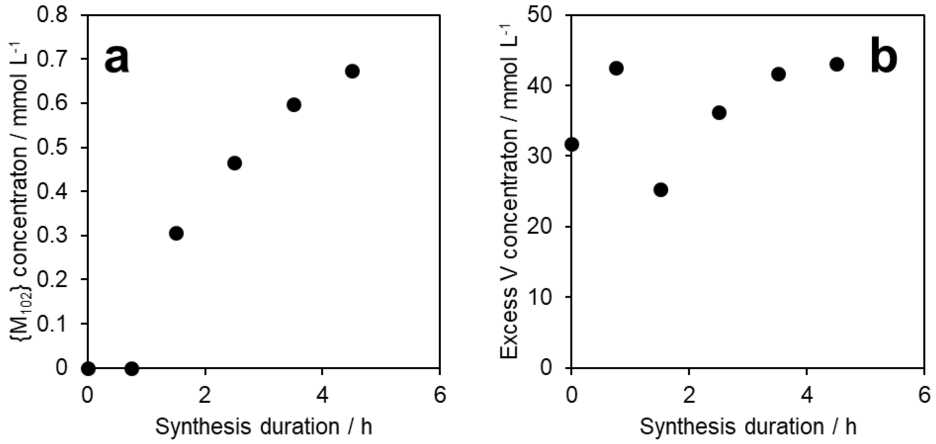

Supplementary Figure 12. Estimated concentrations of {M_{102}} (a) and excess V species (b) in aqueous solution for the new synthesis protocol (values calculated from corresponding metal concentrations shown in Figure 7a). The assumption has been made that all Mo in solution is forming {$Mo_{72}V_{30}$} species.

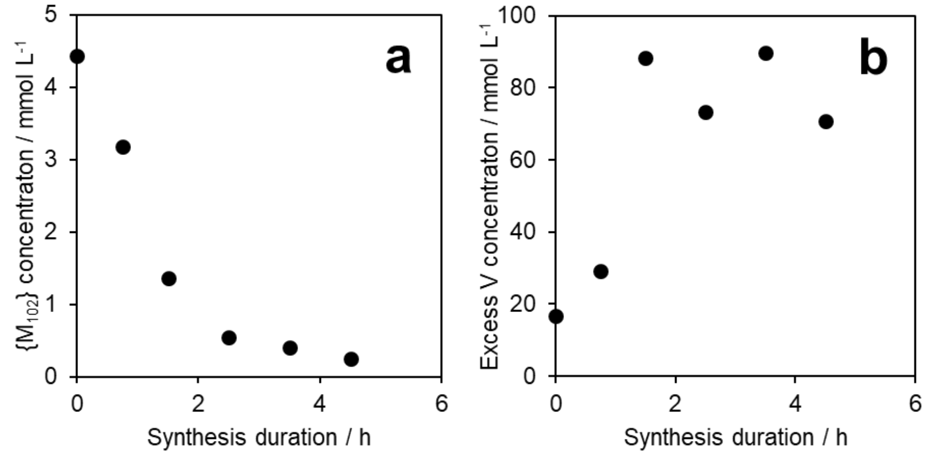

Supplementary Figure 13. Estimated concentrations of {M_{102}} (a) and excess V species (b) in aqueous solution for the standard synthesis protocol according to ref [1] (values calculated from corresponding metal concentrations shown in Figure 7c). The assumption has been made that all Mo in solution is forming {$Mo_{72}V_{30}$} species.

Activity in ethane ODH

Supplementary Figure 14 shows variants of normalization of catalytic ODH-E of MoVTeNbOx samples synthesized for different durations.

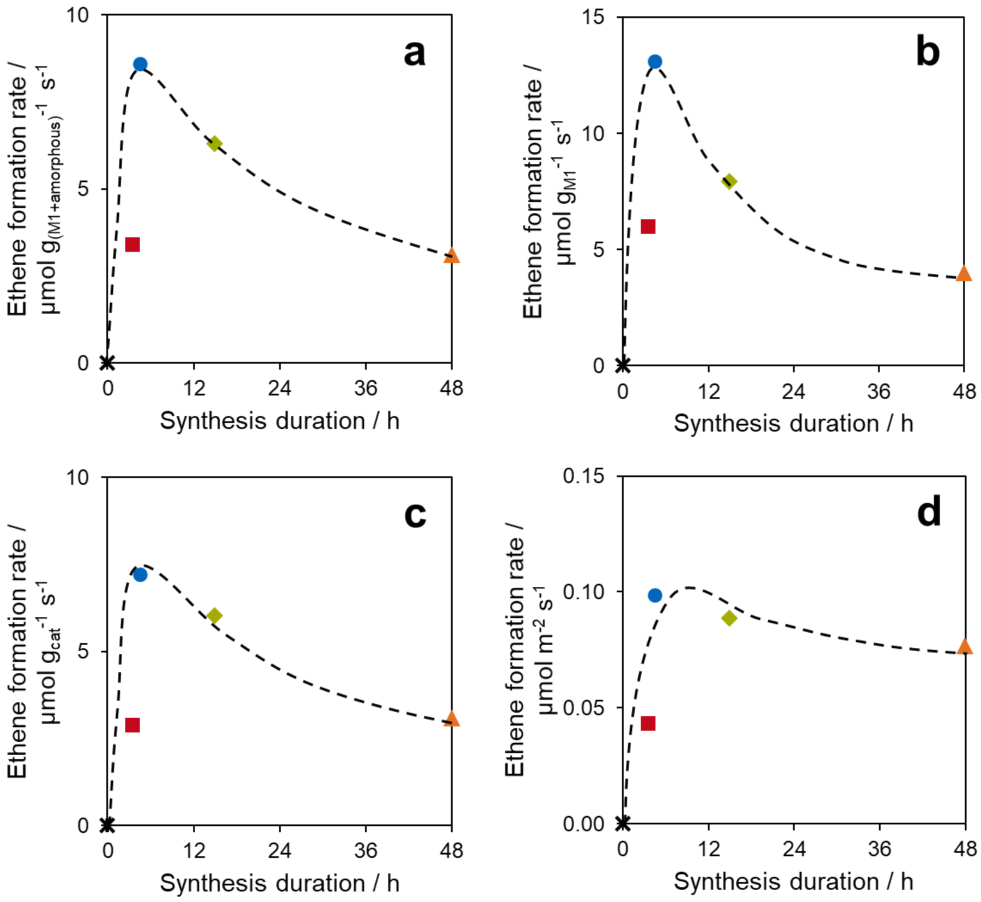

Supplementary Figure 14. Ethene formation rate at 330°C obtained over $MoV_{0.30}Te_{0.05}Nb_{0.05}O_x$ synthesized at 190 °C and 17.5 bar(a) for various durations. T = 330 °C, p = 1 bar(a), WHSV = 7.0 – 13.8 h^{-1}. Rates were normalized to sum of M1 and amorphous phase content (a), mass of M1 phase (b), mass of catalyst used (c) and BET area (d).

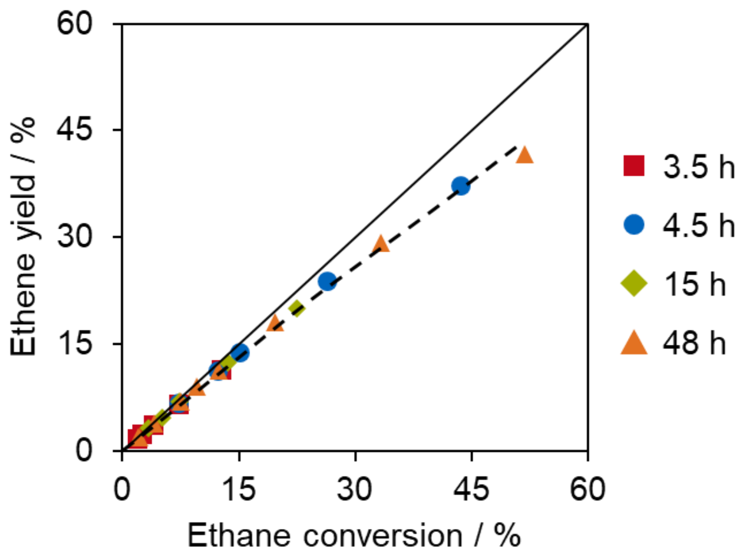

Supplementary Figure 15. Ethene yield as function of ethane conversion obtained over $MoV_{0.30}Te_{0.05}Nb_{0.05}O_x$ synthesized at 190 °C and 17.5 bar(a) for various synthesis durations. T = 330 – 420 °C, p = 1 bar(a), WHSV = 3.6 – 13.8h^{-1}.

Electron microscopy

High angle annular dark field scanning transmission electron microscopy (HAADF-STEM) and scanning electron microscopy (SEM) were applied to study crystal edge termination and morphology of M1 particles. Supplementary Figures 16 to 19 show typical SEM images of $MoV_{0.30}Te_{0.05}Nb_{0.05}O_x$ samples synthesized for different durations.

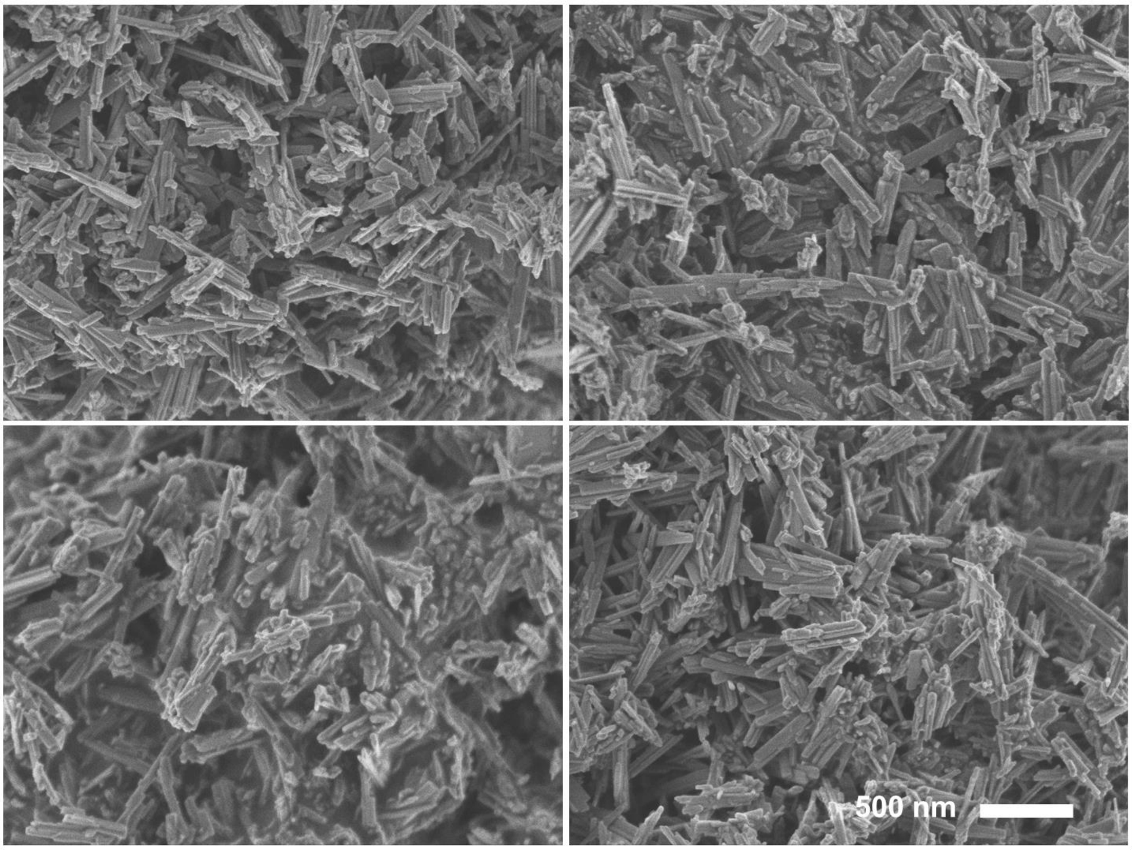

Supplementary Figure 16. SEM images of $MoV_{0.30}Te_{0.05}Nb_{0.05}O_x$ synthesized for 3.5 h according to the new synthesis method and dried overnight at 80 °C in air.

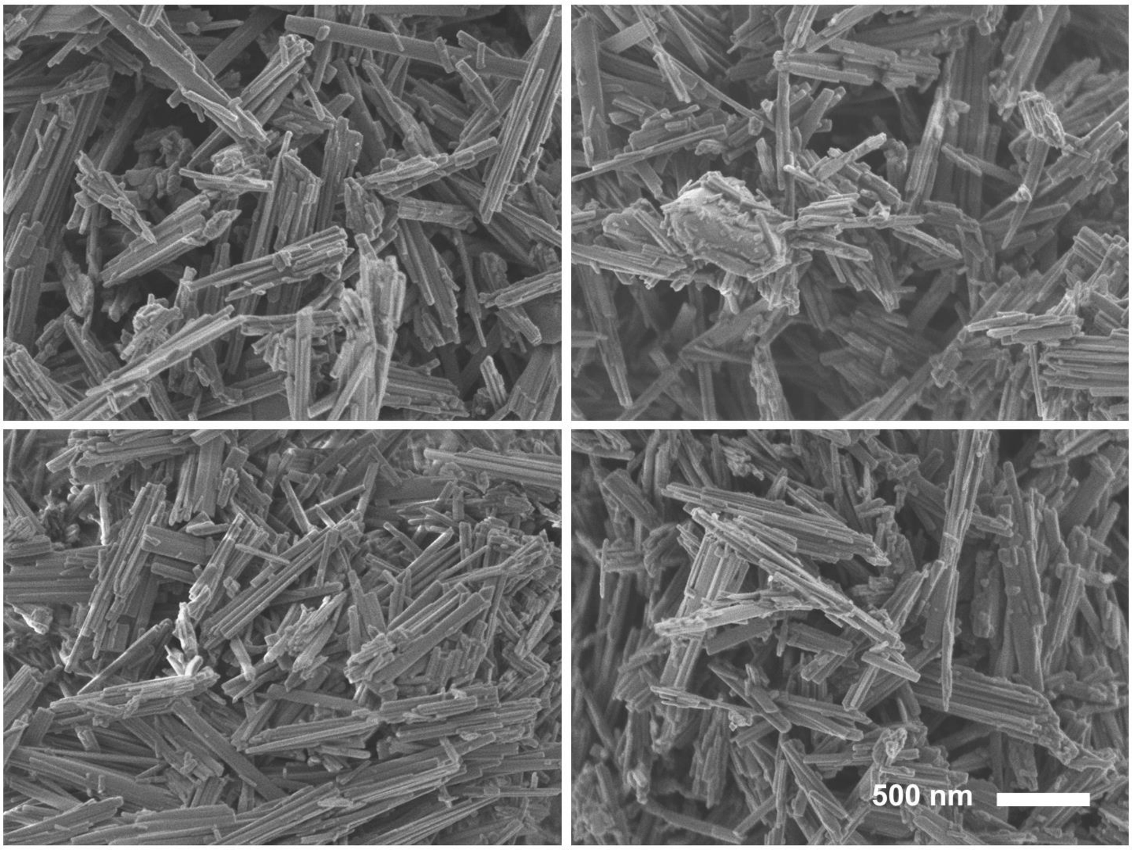

Supplementary Figure 17. SEM images of $MoV_{0.30}Te_{0.05}Nb_{0.05}O_x$ synthesized for 4.5 h according to the new new synthesis method and dried overnight at 80 °C in air.

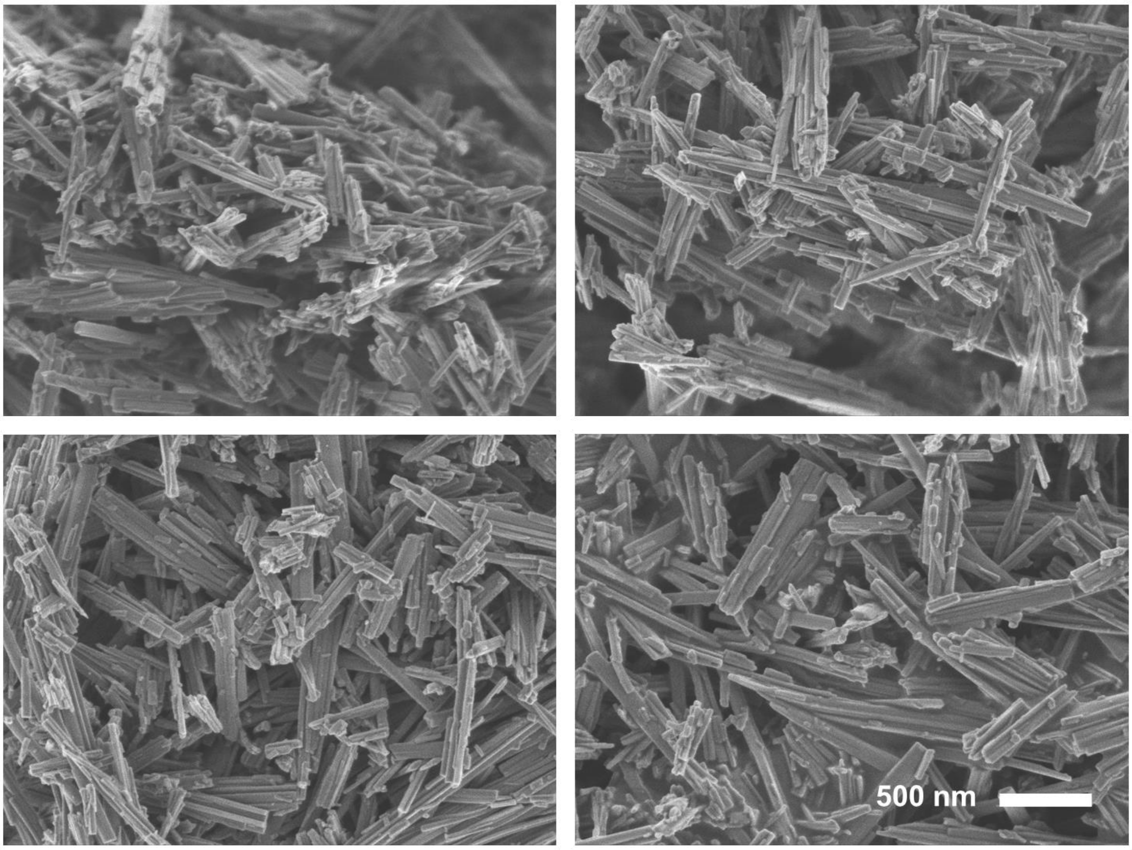

Supplementary Figure 18. SEM images of $MoV_{0.30}Te_{0.05}Nb_{0.05}O_x$ synthesized for 15 h according to the new new synthesis method and dried overnight at 80 °C in air.

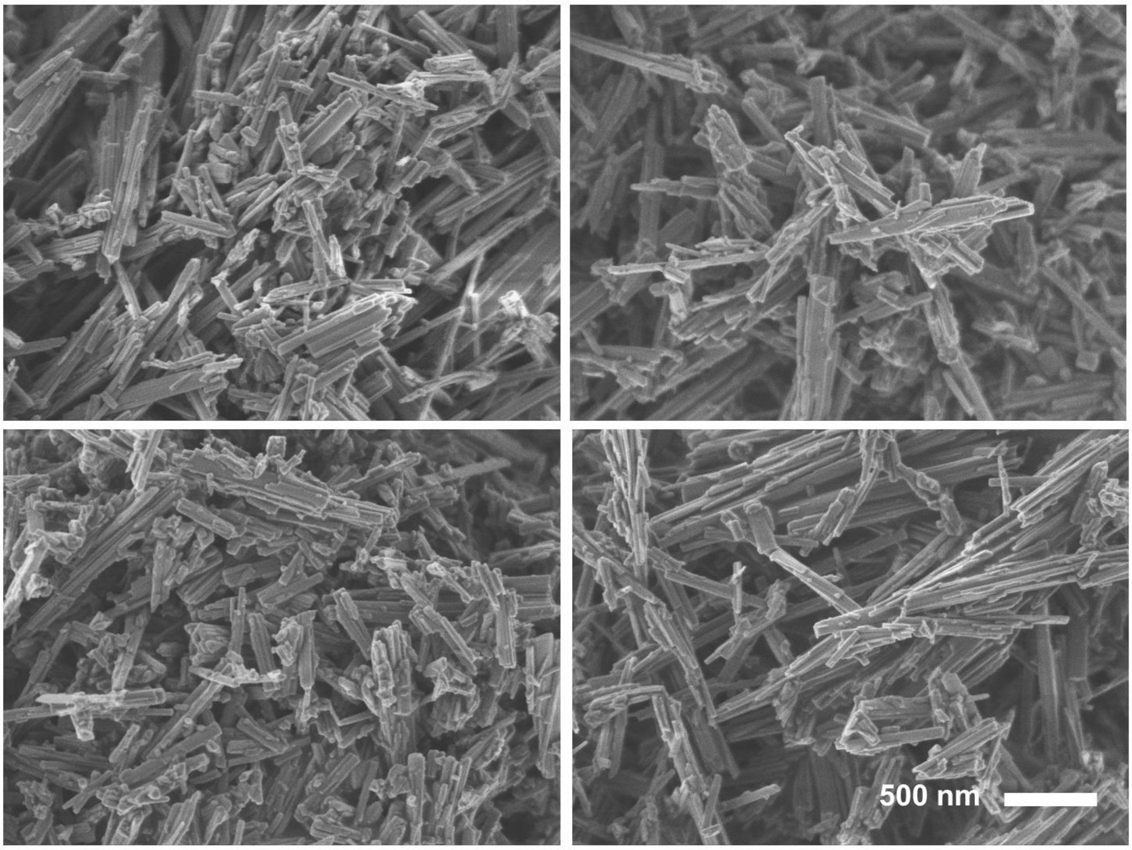

Supplementary Figure 19. SEM images of $MoV_{0.30}Te_{0.05}Nb_{0.05}O_x$ synthesized for 48 h according to the new new synthesis method and dried overnight at 80 °C in air.

Supplementary Figure 20 shows the particle size distribution of $MoV_{0.30}Te_{0.05}Nb_{0.05}O_x$ synthesized for various durations along the crystals longest axis ([001] direction). Size distribution was derived from SEM images by counting of more than 300 particles per sample.

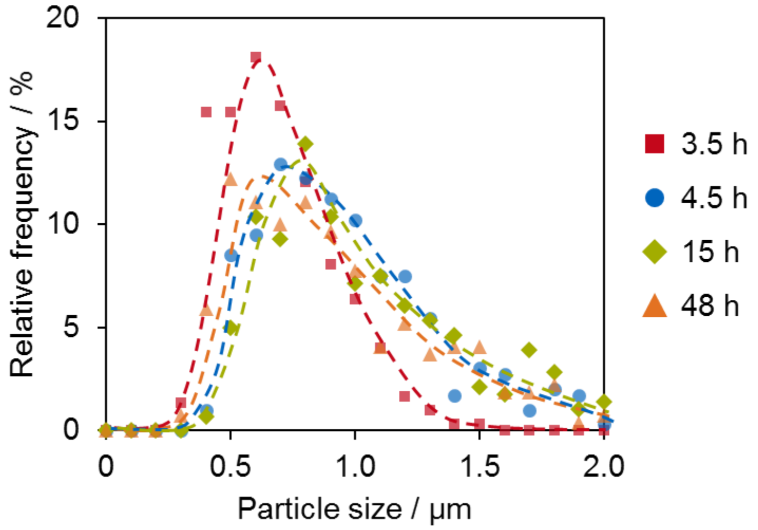

Supplementary Figure 20. Particle length distribution of $MoV_{0.30}Te_{0.05}Nb_{0.05}O_x$ synthesized for various durations according to the new new synthesis method and dried overnight at 80 °C in air.

Although quantitative analysis of the size of the cross section of the needles is not possible, visual inspection of SEM images in Supplementary Figures 16-19 leads us to conclude that the diameter-length aspect ratio does not change for synthesis times above 3.5 h.

For the sake of comparison, SEM images of a MoVTeNbOx material prepared by the standard method and crystallized at 650 °C were also taken (Supplementary Figure 21). The M1 particles prepared in this way show a somewhat larger diameter/length aspect ratio and an overall shorter particle length (Supplementary Figure 22).

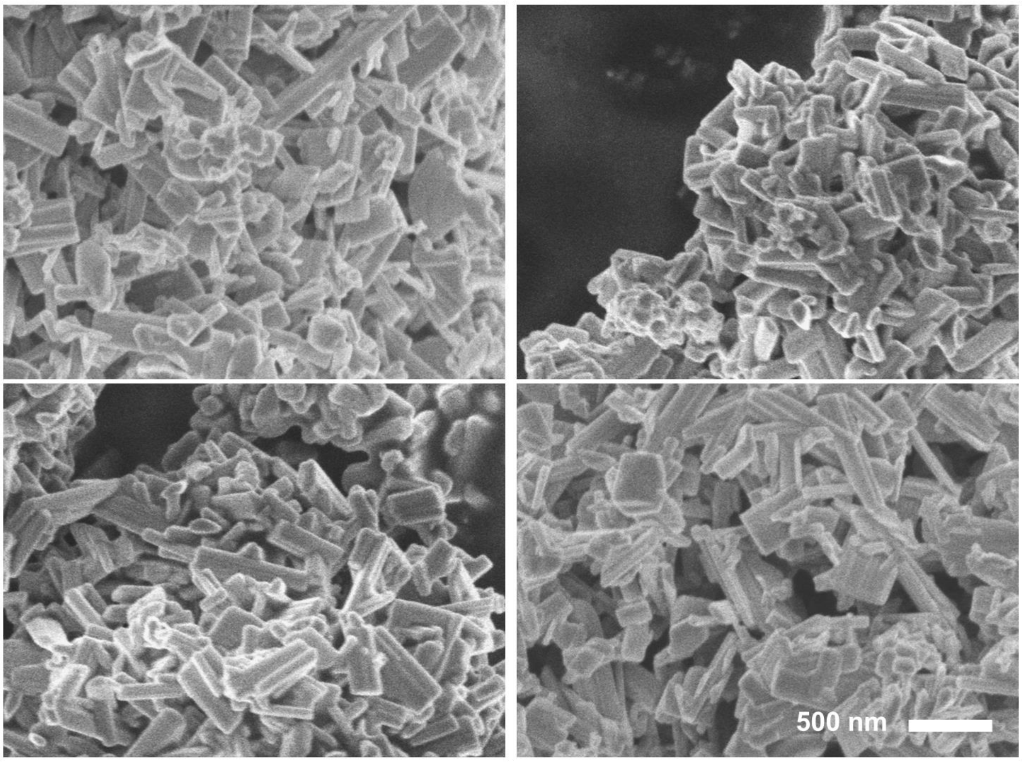

Supplementary Figure 21. SEM images of $MoV_{0.30}Te_{0.06}Nb_{0.08}O_x$ standard material synthesized according to the method presented in reference [1] and crystallized at 650 °C.

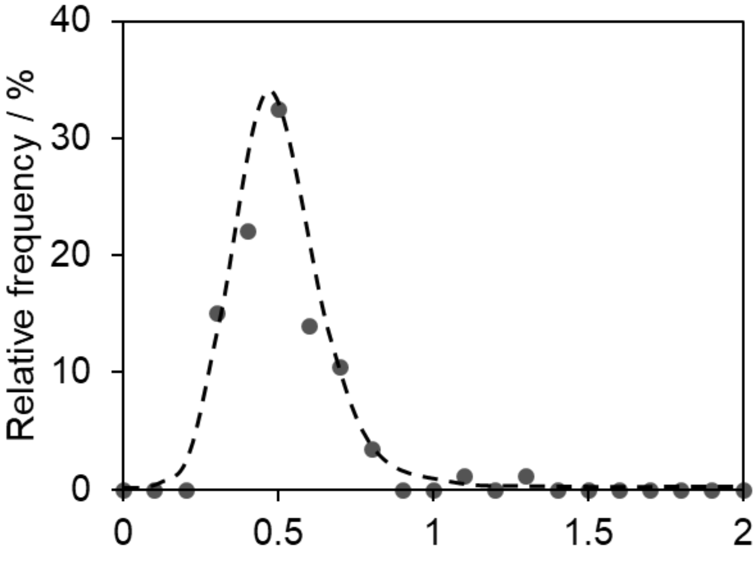

Supplementary Figure 22. Particle length distribution of $MoV_{0.30}Te_{0.06}Nb_{0.08}O_x$ standard material synthesized according to the method presented in reference [1] and crystallized at 650 °C.

Supplementary Figures 23 – 26 show HAADF-STEM micrographs of $MoV_{0.30}Te_{0.05}Nb_{0.05}O_x$ samples of different hydrothermal synthesis duration corresponding to the SEM images in Supplementary Figures 16-19.

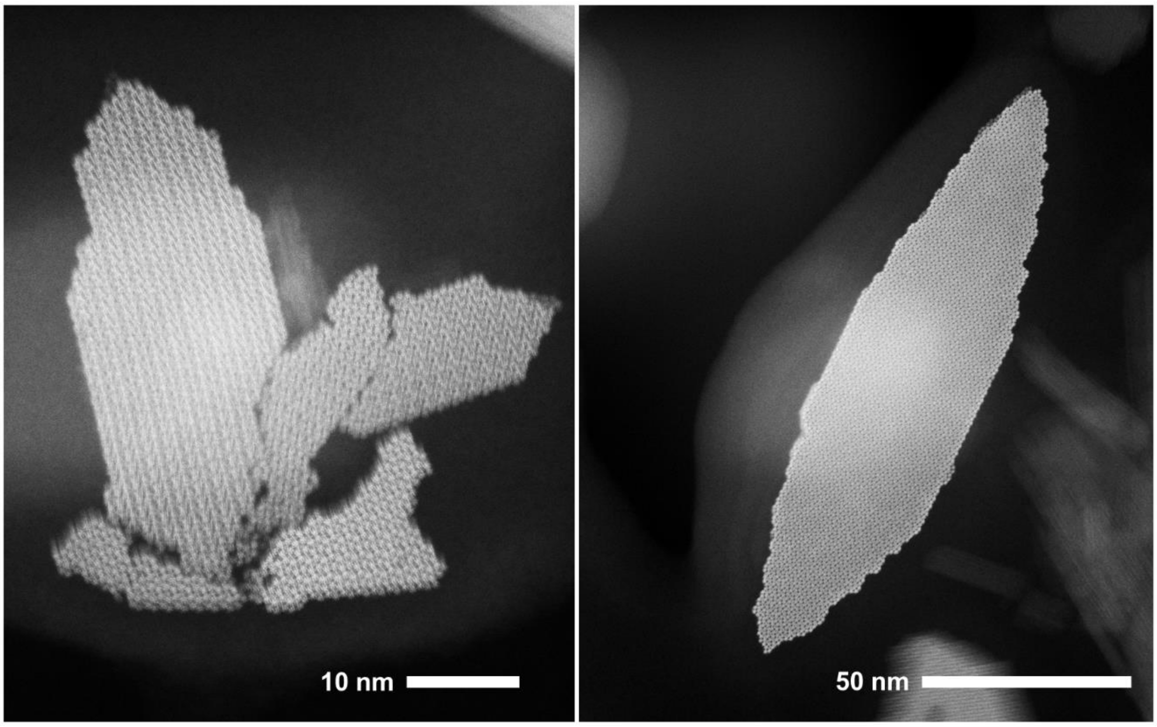

Supplementary Figure 23. HAADF.STEM images of $MoV_{0.30}Te_{0.05}Nb_{0.05}O_x$ synthesized for 3.5 h according to the new synthesis method and dried overnight at 80 °C in air.

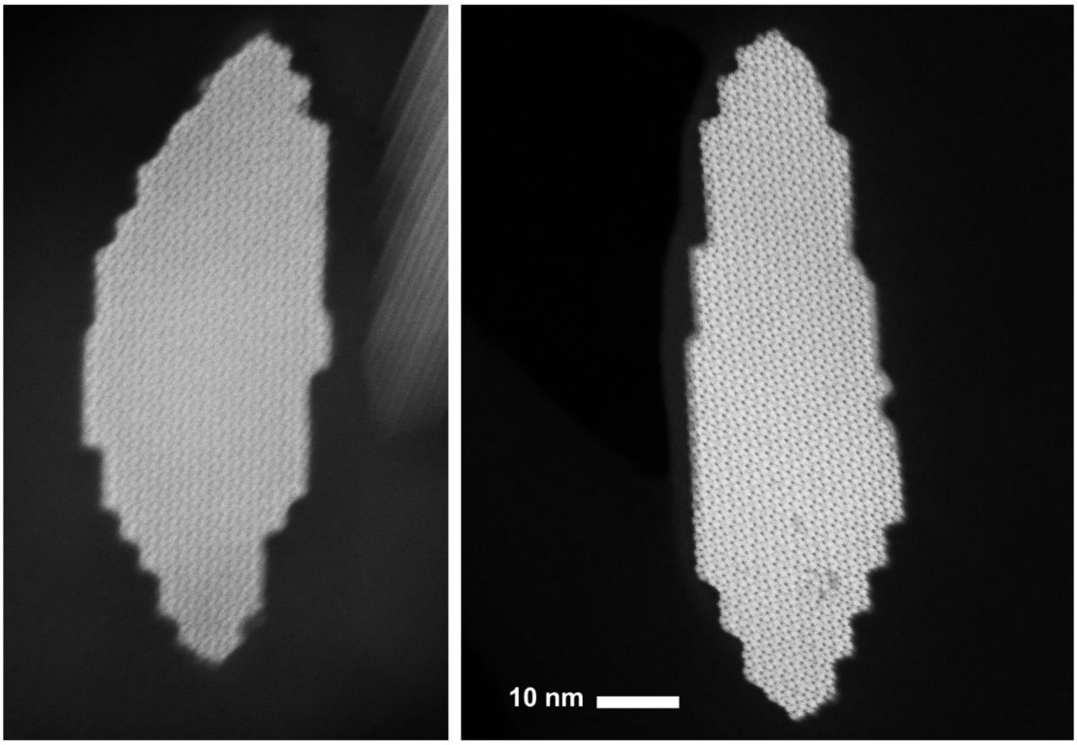

Supplementary Figure 24. HAADF.STEM images of $MoV_{0.30}Te_{0.05}Nb_{0.05}O_x$ synthesized for 4.5 h according to the new synthesis method and dried overnight at 80 °C in air.

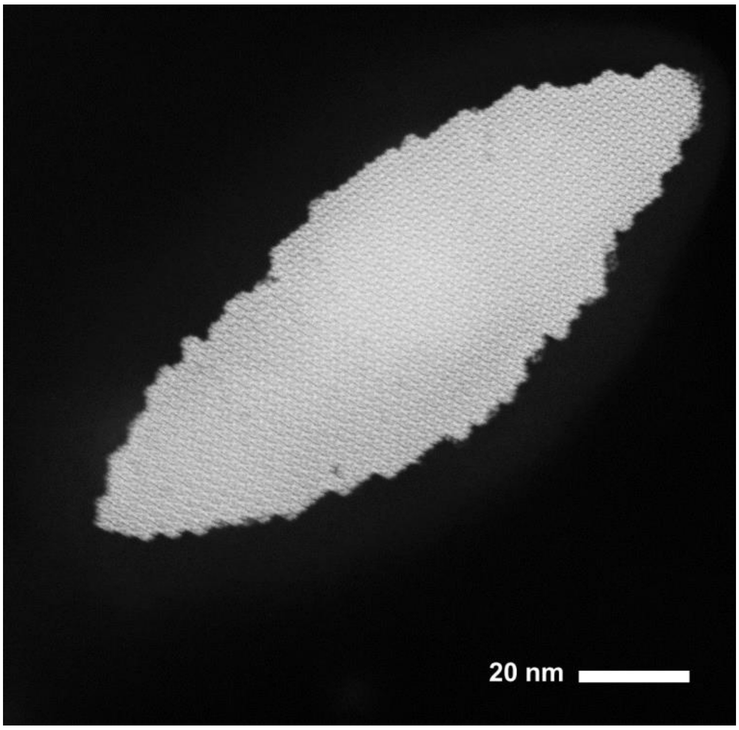

Supplementary Figure 25. HAADF.STEM image of $MoV_{0.30}Te_{0.05}Nb_{0.05}O_x$ synthesized for 15 h according to the new synthesis method and dried overnight at 80 °C in air.

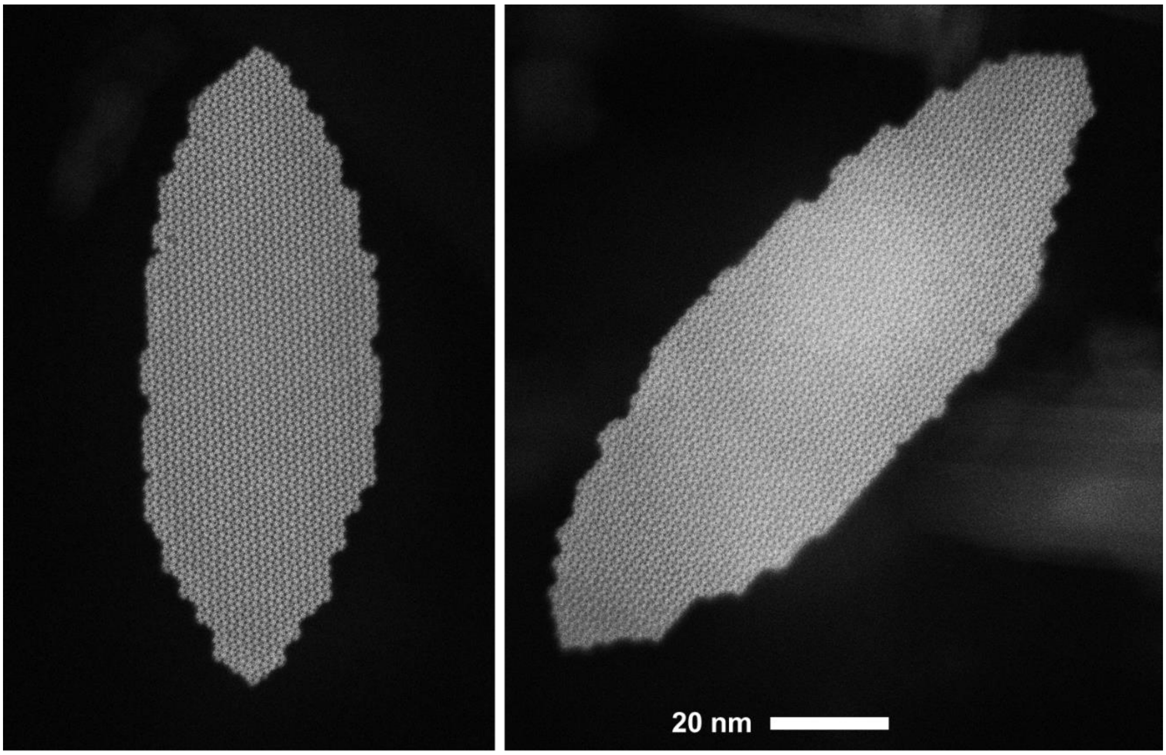

Supplementary Figure 26. HAADF-STEM images of $MoV_{0.30}Te_{0.05}Nb_{0.05}O_x$ synthesized for 48 h according to the new synthesis method and dried overnight at 80 °C in air.

Supplementary References

1. Celaya Sanfiz A*, et al.* Preparation of Phase-Pure M1 MoVTeNb Oxide Catalysts by Hydrothermal Synthesis—Influence of Reaction Parameters on Structure and Morphology. *Topics in Catalysis* **50**, 19-32 (2008).
